# Herd Routines and Veterinary Advice Related to Dry-Cow Therapy and Treatment with Internal Teat Sealants in Dairy Cows

**DOI:** 10.3390/ani11123411

**Published:** 2021-11-29

**Authors:** Karin Persson Waller, Håkan Landin, Ann-Kristin Nyman

**Affiliations:** 1 Department of Animal Health and Antimicrobial Strategies, National Veterinary Institute (SVA), SE-75189 Uppsala, Sweden; 2 District Veterinarians, Board of Agriculture, SE-84631 Hede, Sweden; hakan.landin@distriktsveterinarerna.se; 3 Department of Animal Health and Development, Växa Sverige, SE-10425 Stockholm, Sweden; ann.nyman@vxa.se; 4 Department of Clinical Sciences, Swedish University of Agricultural Sciences (SLU), SE-75007 Uppsala, Sweden

**Keywords:** mastitis, drying-off, dry-cow therapy, internal teat sealants, dairy cows, intramammary antibiotics, Sweden

## Abstract

**Simple Summary:**

At the end of lactation, antibiotics (DCT) or internal teat sealants (ITS) can be used to treat or prevent mastitis in dairy cows. Recommendations on how to perform such treatments are available, but little is known about how well these are followed by farmers and veterinarians. To increase this knowledge, questionnaires about farmer routines and veterinary advice were sent to 2472 farmers and 517 veterinarians in Sweden. Fourteen percent of the farmers and 25% of the veterinarians responded. Among the farmers, 81% used DCT to some cows, 3% used DCT to all cows, and 16% did not use DCT at all. Almost all veterinarians prescribed DCT, most only to some cows in a herd while 8% sometimes recommended DCT to all cows in a herd. Most of the farmers did not use ITS and half of the veterinarians never prescribed ITS. Milking system and milk production, and post-graduate training and number of mastitis cases per month were associated with several of the answers by the farmers and veterinarians, respectively. Overall, many farmers and veterinarians followed the recommendations, but it was also clear that more communication is needed as well as an up-date of the recommendations.

**Abstract:**

Dry-cow therapy with antibiotics (DCT) and treatment with internal teat sealants (ITS) are often used to control mastitis in dairy cows. However, the knowledge on farmer and veterinary compliance with recommendations for DCT and ITS is scarce. Thus, the main aim was to collect information on farmer routines and veterinary advice for such treatments. Associations with herd and veterinary variables were also studied. Web-based questionnaires including questions on demographics and the use of DCT and ITS were sent to 2472 farmers and 517 veterinarians in Sweden. The answers were summarized descriptively, and associations with demographics were evaluated using univariable regression models. The response rate was 14% for farmers and 25% for veterinarians. Among the farmers, 81% used selective DCT (SDCT), 3% used blanket DCT (BDCT), and 16% did not use DCT. Almost all (93%) veterinarians prescribed DCT and among those most recommended SDCT while 8% recommended BDCT. Eighty-two percent of the farmers did not use ITS and 45% of the veterinarians never prescribed ITS. Milking system and milk production, and post-graduate training and number of mastitis cases per month were associated with the largest numbers of farmer and veterinary answers, respectively. In conclusion, many farmer routines and veterinary advice complied with the recommendations available at the time, but a clear need for more education was also identified. The results also indicated that an up-date of the national recommendations was warranted.

## 1. Introduction

Dry-cow therapy with antibiotics (DCT) has been a part of control programmes for mastitis in dairy cows in many countries of the world for more than 50 years, as reviewed by [1]. In many countries blanket DCT (BDCT), i.e., treatment of all cows at drying-off has been recommended. In the Nordic countries, however, selective DCT (SDCT), i.e., only treating cows with infectious subclinical mastitis, has always been the norm as outlined recently by Rajala-Schultz et al. [2]. When using SDCT it is important to have good routines for selection of cows to ensure that only cows with a good prognosis for treatment success are treated. Recommendations on DCT have been available from the main Swedish advisory organization (Växa Sverige (former Swedish Dairy Organization), Stockholm, Sweden (www.vxa.se), accessed on 29 November 2021) for dairy farmers for many years. Moreover, such recommendations are also included in the Swedish national guidelines for veterinary use of antibiotics [3], and some aspects of the use of DCT are regulated in the national legislation for veterinarians [4]. In addition, in the Nordic countries, drugs for DCT can only be prescribed by a veterinarian. Recommendations on DCT are also available in other countries [5,6,7,8], but there is no consensus on the best routine to perform DCT. Recently, however, the interest for SDCT and for finding the best tools to use when selecting cows suitable for such treatment has increased e.g., [9,10,11].

In many countries, the use of internal teat sealants (ITS) to all, or a selection of cows, is also recommended at drying-off to reduce the risk of new intramammary infections (IMI) during the dry period reviewed by [12]. ITS has been available in Sweden for some time, but if and how often these products are used is not known. Moreover, as for DCT, ITS can only be prescribed by a veterinarian, and the use of ITS have so far not been included in the recommendations to farmers and guidelines for veterinarians.

To our knowledge, detailed investigations of routines for DCT and ITS used by commercial farmers are scarce [13], and none has been performed in Sweden. Thus, it is not known if Swedish farmers follow the recommendations from the Swedish advisory organization or not. Moreover, it is not known if field veterinarians working with dairy cattle use those recommendations when advising farmers. In addition, the attitudes of farmers and veterinarians to the importance of DCT and ITS to animal health and production are not known.

Thus, the main aims of this study were to collect information on farmer routines and attitudes as well as on veterinary advice on DCT and ITS using web-based questionnaires. In addition, we wanted to investigate if routines and advice were associated with herd and veterinary variables. The long-term goal was to evaluate the need for more education and an update of recommendations.

## 2. Materials and Methods

Two web-based anonymous questionnaires, one for farmers and one for veterinarians, were produced (Questback Essentials, Stockholm, Sweden). The farmer questionnaire was modified from the questionnaire used by Vilar et al. [13]. The questionnaires (in Swedish) are provided in the Supplementary Documents S1 and S2. The contents and quality of the questionnaires were tested by a small group of farmers and veterinarians before performing the study.

The questionnaires included several sections, the first contained demographic questions about the herd (number of cows/year, county, conventional or organic production, average annual milk production per cow, average estimated bulk milk somatic cell count (SCC), milking system), or the veterinarian (year of veterinary degree, country of veterinary degree, county, gender, post-graduate training in bovine diseases, number of years in cattle practice, number of mastitis cases/month). The other parts contained questions on drying-off, dry cow therapy using antibiotics, treatment with internal teat sealants, and the dry period. The questions on drying-off and the dry period will be presented in a separate publication. Some questions were mandatory and some questions resulted in additional questions depending on the answers. In both questionnaires, DCT was defined as infusing antibiotics (mostly long-acting) via the teat canal into all udder quarters after the last milking during drying-off, i.e., just before the start of the dry period, and treatment with ITS was defined as infusing bismuth subnitrate (not antibiotics) via the teat canal into all udder quarters after the last milking during drying-off, i.e., just before the start of the dry period.

The questionnaires were sent to the target groups in the end of 2019/beginning of 2020 (late autumn/early winter). Information about the questionnaire and a link to the website was distributed to the farmers via an email sent to all Swedish dairy producers having an email address and being affiliated to one of the dairy cattle farmers organisations Växa Sverige, Skånesemin or Rådgivarna i Sjuhärad (*n* = 2472 representing 75% of all Swedish dairy farmers (Jordbruksverket 2019)). Information about the questionnaire and a link to the website was distributed to the veterinarians via an e-mail sent to all veterinarians born in 1950 or later that had treated at least one case of bovine mastitis during 2018 and were registered with an email address at the National Board of Agriculture (*n* = 530). The email addresses of 43 veterinarians were not valid so the questionnaire reached 487 veterinarians. The same questionnaire was also sent via email to all veterinarians (*n* = 30) employed at the three dairy cattle farmers organisations mentioned above. Thus, the questionnaire was sent to 517 veterinarians. Both questionnaires were open for 4 weeks and reminders were sent via e-mail approximately once a week.

The national recommendations for DCT available at the time of the study can be summarized as follows: Cows for DCT are selected based on udder health class (UHC). The UHC is provided by the Swedish official milk recording scheme (Kokontrollen, Växa Sverige, Stockholm) and is calculated from the cow SCC (CSCC) at 2–3 consecutive milk recordings using regression analysis [14]. The UHC at the last milk recording before drying-off is used for selection of cows as follows; UHC 0–2 (average CSCC < 130,000 cells/mL) = no DCT (healthy), UHC 3–8 (average CSCC 130,000 to 600,000 cells/mL) = DCT is decided based on SCC and growth of bacteria, UHC 9 (average CSCC > 600,000 cells/mL) = no treatment sue to poor prognosis. Only cows with subclinical mastitis due to IMI sensitive to penicillin are selected. All four udder quarters are treated with long-acting antibiotics after the last milking during drying-off after thorough cleaning of the teat end (with alcohol). At the time of the study two long-acting DCT products were available in Sweden, Benestermycin^®^ (Boehringer Ingelheim Animal Health Nordics, Malmö, Sweden) containing benetamine penicillin, penetamate hydroiodide and framycetin sulphate, and Siccalactin^®^ (Boehringer Ingelheim Animal Health Nordics, Malmö, Sweden) containing benzyl penicillin benzatine and dihydrostreptomycin. Teat dip/spray immediately after treatment and check the udder and teat dip/spray morning and evening for 24 h after treatment. If the herd has no access to UHC, CMT results can be used to control udder health. Given the long withdrawal time of the products the time to estimated calving should be at least 6 weeks if long-acting antibiotics are used. All DCT cows should be checked with CMT after calving and control of CSCC at first milk recording, and milk samples for bacteriology should be taken if CMT > 2 (scale 1–5) or CSCC is above 150,000 cells/mL. At the time, there were no Swedish recommendations for ITS available.

To ensure sufficient numbers to perform valid statistics farmers or veterinarians in each geographical area the counties (*n* = 21) were compiled into regions according to Nomenclature of Territorial Units for Statistics (NUTS) level 2 with two modifications: Middle and Upper Norrland were combined into Norrland, and Stockholm and East Middle Sweden were combined into East Sweden. This resulted in six geographical regions, i.e., Norrland, North Middle Sweden, East Sweden, Småland and the islands, West Sweden, and South Sweden. The cut-offs for the continuous variables: number of cows/years, year of veterinary degree, and number of years in cattle practice were set to give approximately equal number of observations per category. The categories for the other continuous variables: milk production, bulk milk SCC, and number of mastitis treatments per month were pre-set in the questionnaire. Three categories for the bulk milk SCC were used (<200,000 cells/mL, 200,000 to 300,000 cells/mL, and >300,000 cells/mL) but the number of herds having >300,000 cells/mL were very few. Thus, only two categories were used in the analyses, i.e., <200,000 cells/mL and ≥200,000 cells/mL.

The answers to the questionnaires were summarized descriptively. Statistical differences within each target group based on information on herds and veterinarians were evaluated using univariable logistic or multinomial logistic regression models when questions had enough observations per outcome and answer category. In the models, the answers in the questionnaires were treated as outcomes and the demographic variables as explanatory variables. Due to the vast number of univariable analyses, no multivariable analyses were performed. The herd variables (categories) used were region (six regions as specified above), production type (conventional, organic), milking system (automatic milking system (AMS), tied-up, parlour, rotary, combinations), number of cows/year (<53, 53–77, 78–137, ≥138 cows), average yearly milk production per cow (<9000 kg energy corrected milk (ECM), 9000–11,000 kg ECM, >11,000 kg ECM), and estimated bulk milk SCC (<200,000 cells/mL, ≥200,000 cells/mL). Housing of lactating cows was omitted from the analyses due to the categories being very similar to milking system categories, where the housing system was included. The veterinary variables (categories) were year of veterinary degree (1977–1991, 1992–2001, 2002–2008, 2009–2014, 2015–2020), country of veterinary degree (Sweden, Denmark/Norway/Finland, other countries in Europe), region (six regions, as specified above), gender (female, male), post-graduate training in bovine diseases (yes, no), type of post-graduate training (Hälsopaket mjölk, ViLA, other), number of years in cattle practice (<5, 5–9, 10–14, 15–19, 20–24, ≥25 years) and number of mastitis treatments per month (<1, 1–3, 4–8, 9–15, >15 cases).

## 3. Results

The questionnaire response rate was 14% (340 of 2472) for farmers and 25% (130 of 517) for veterinarians. The results are presented descriptively below followed by results from the statistical analyses when relevant.

### 3.1. Herd and Veterinary Variables

Descriptive information on the respondents is given in Table 1 and Table 2. In short, most dairy herds were situated in West Sweden or Småland and the islands, had conventional production and housed their dairy cows in insulated free-stall buildings. Automatic milking system (AMS) was the most common milking system and the average number of cows per herd and year was 116 (median 78). Most herds produced 9000 to 11,000 kg ECM/year and had an annual average bulk SCC below 200,000 cells/mL.

Among the veterinarians, the year of veterinary degree varied from 1977 to 2020, with a median of 2005. Most of them obtained their degree in Sweden, and worked in East Sweden, Norrland, or West Sweden. The majority was female with some type of post-graduate training in cattle diseases. The number of years in cattle practice varied markedly (0–39 years; median 10–14 years) as did the number of mastitis cases per month (<1 to >15 cases; median 4–8 cases/month).

### 3.2. Questions about DCT to Farmers

Descriptive statistics on DCT routines used by the farmers are given in Table 3. Below a short description of the results is given.

#### 3.2.1. Use of DCT and Selection of Cows

Eighty-one percent of the herds used SDCT, mainly treating the occasional cow or fewer than 25% of the cows, and 3% used BDCT. Farmers who did not use DCT (16% of the herds) stated good udder health and/or concern for antimicrobial resistance as the main reasons for not doing so.

Most of the SDCT farmers selected cows using UHC, the CSCC at the last milk recording before drying-off, and/or the occurrence of clinical mastitis during the previous lactation. Milk samples for bacteriological examination were taken before deciding on DCT from some of the cows in half of the herds.

#### 3.2.2. Choice of Antimicrobials

Most herds (55%) only used Siccalactin^®^ while 22% only used Benestermycin^®^. Remaining herds used both of those two products, with no preference to one or the other, and a few herds used Carepen^®^ (Boehringer Ingelheim Animal Health Nordics, Malmö, Sweden) an intramammary product only containing short-acting benzyl penicillin procaine. Most (84%) farmers always treated all four udder quarters, while the rest stated that they only treat lactating udder quarters or those with high CMT.

#### 3.2.3. Preparing for and Administering Treatments

Around half of the farmers stated that they clean their hands before treatment and 40% used clean gloves. Almost all farmers used the same routine at the actual treatment, i.e., wiping with serviette provided and full insertion of the tube tip into the teat canal. Most (71%) did not think there were any risks or difficulties with the treatment but 11% stated that kicking of the cow is a risk and 11% that there is a risk to introduce dirt/bacteria into the teat.

#### 3.2.4. Assessment of Treatment Effects

The effects of the DCT were always/almost always evaluated by 58% of the farmers and 19% stated that they rather often examined the CSCC at the first milk recording after calving. CMT was always/almost always and rather often performed in 27% and 18% of the herds, respectively. Milk sampling for bacteriology after calving was rare. Almost all farmers thought that DCT improves cow udder health in the beginning of the next lactation while 64% and 63% thought that the milk production and cow longevity, respectively, improves. Approximately one-fourth of the farmers were uncertain about the effects of DCT on those parameters. Just over half of the farmers did not know if calf health is affected by DCT while 35% did not think it had any effect.

### 3.3. Associations between Demographic Variables and Answers Given by Farmers

As can be seen in Table 3, all six herd variables, but especially milking system and milk production, were associated with the responses given by the farmers. Detailed information on the results is given in Supplementary Table S1. Here, only a short summary with some examples is given.

#### 3.3.1. Use of DCT and Selection of Cows

It was less common to use DCT in herds producing <9000 kg ECM or having <53 cows than in herds with higher milk production and ≥138 cows, respectively. In herds with a BMSCC ≥200,000 cells/mL it was more common to state poor udder health as the reason for using DCT while herds producing ≥9000 kg ECM more often stated recommendation from an advisor as the reason than in herds producing less milk.

In herds using SDCT and producing ≤11,000 kg ECM, it was more common to treat the occasional cow, compared to in herds with higher production where it was more common to treat at least 25% of the cows. In SDCT herds, it was also more common to treat at least half of the cows, compared to the occasional cow, in herds with a BMSCC ≥200,000 cells/mL than in herds with lower BMSCC. A larger proportion of cows was treated in herds with AMS than in herds with tie-stall milking, and in herds with ≥53 cows than in herds with fewer cows.

#### 3.3.2. Choice of Antimicrobials

The selection of DCT product differed between regions, for example it was more common to use Benestermycin^®^ in West Sweden than in East Sweden and more common to use Siccalactin in Norrland than in Småland and the islands. Benestermycin^®^ was also more commonly used in the highest producing herds while Siccalactin^®^ was more common in herds with lower production.

#### 3.3.3. Preparing for and Administering Treatments

To wash hands before DCT was more common in herds with AMS or tie-stall milking than in herds with parlour milking. To use clean gloves at DCT was more common in herds producing ≥9000 kg ECM and in herds with milking parlour than in herds with lower production and herds with tie-stall milking or AMS, respectively. Wiping the teats with paper was more common in organic than in conventional herds while wiping with a moist single-use towel was more common in herds with tie-stall milking or parlour than in AMS herds. The use of full insertion of the tip of the intramammary tube was more common in some regions than in others. More herds with organic production stated that there are risks or difficulties with the treatment.

#### 3.3.4. Assessment of Treatment Effects

The proportion of herds that evaluated the effects of DCT by examining the CSCC at the first milk recording varied with the number of cows at the herd. Overall, it was more common to always do so in smaller herds. To evaluate the effects of DCT by CMT after calving was more common in herds with a BMSCC < 200,000 cells/mL than in herds with higher BMSCC and more common in herds with parlour milking or tie-stall milking than in herds with AMS.

### 3.4. Questions about DCT to Veterinarians

Descriptive statistics on advice given by veterinarians to dairy farmers and/or their personnel on DCT are given in Table 4. Below, a short description of the results is given.

#### 3.4.1. Prescriptions of DCT and Selection of Cows

Almost all veterinarians prescribed DCT, most commonly a few times per month or more. It was not common to regularly use bacteriological investigation of milk samples before prescribing DCT. However, when this was done, it was most common to use an accredited laboratory. Almost all veterinarians stated that they only prescribe one type of DCT product and Siccalactin^®^ was most common. Veterinarians prescribing DCT mainly did so to some cows in the herds, only 8% stated that they sometimes recommend BDCT. The reason for prescribing BDCT was mostly that the herd had problems with *Streptococcus agalactiae.*

The UHC at the last milking before drying-off was the most common factor used when selecting cows for DCT. Other factors influencing the selection were bacterial growth in milk samples, clinical mastitis during lactation, CSCC at the last milk recording, and positive CMT reaction at drying-off. Almost all veterinarians recommended treatment of all four udder quarters. Those who did not, recommended treatment only of quarters with high CMT or lactating quarters.

#### 3.4.2. Giving Advice

Almost two-thirds of the veterinarians did sometimes or often give advice about how to perform the actual treatment. Among those who did not give such advice most stated that they did not perceive an interest for this or did not have enough knowledge as reasons.

#### 3.4.3. Preparing for and Administrating Treatments

When asked which of the presented routines they thought should be a part of a good treatment routine use clean gloves and to wipe the teat end with provided serviettes were the most common answers followed by washing hands before treatment and massage of teat/quarter after infusion of the product. Close to half of the veterinarians recommended full insertion of the tip of the tube while just over one-quarter recommended partial insertion. Most veterinarians thought that there are risks or difficulties with the treatment; poor hygiene/risk to introduce bacteria and risk for antimicrobial resistance being the most common risks considered.

#### 3.4.4. Legislation

One-third of the veterinarians stated that they did not know about the national legislation on DCT. Among those who prescribed DCT half stated that they always/almost always, and one-third that they often, follow the legislation.

#### 3.4.5. Assessment of Treatment Effects

To evaluate the effects of DCT by CMT after calving and by examining CSCC at the first milk recording were common recommendations. Almost all veterinarians thought that DCT improves cow udder health in the beginning of the coming lactation while around 60% thought it improves milk production and cow longevity. Almost half of the veterinarians stated that they did not know if DCT affects calf health and around one-fourth did not know if DCT affects milk production and cow longevity.

### 3.5. Associations between Demographic Variables and Answers Given by Veterinarians

As can be seen in Table 4, all veterinary variables, but especially post-graduate training and number of mastitis cases per month, were associated with the responses given by the veterinarians. Detailed information on the results is given in Supplementary Table S2. Here, a summary of the results with some examples is given.

#### 3.5.1. Prescriptions of DCT and Selection of Cows

The frequency of prescribing DCT varied between regions and was, for example, higher in South and West Sweden than in North Middle Sweden. Veterinarians with post-graduate training in cattle diseases prescribed DCT more often than those without training. Moreover, veterinarians treating more mastitis cases per month prescribed DCT more often than those with fewer cases. Veterinarians working in Norrland performed bacteriological culturing themselves more often than veterinarians in Northern Middle Sweden and South Sweden. It was more common that veterinarians treating relatively few mastitis cases per month sent milk samples to an accredited laboratory than veterinarians treating many cases. The preferred DCT product varied between regions. For example, veterinarians in West Sweden more often prescribed Benestermycin^®^ than veterinarians in Norrland. More veterinarians with post-graduate training prescribed Benestermycin^®^ than those without training. It was also more common to prescribe Benestermycin^®^ among veterinarians treating more than eight mastitis cases/month than among those treating fewer cases.

Veterinarians with post-graduate training in cattle diseases more often recommended DCT to the occasional cow than veterinarians without training. Veterinarians without post-graduate training more often stated that a CMT reaction in an udder quarter affected the selection of cows for DCT than veterinarians with training

#### 3.5.2. Giving Advice

It was more common among veterinarians with post-graduate training or treating relatively more mastitis cases to give advice on how to perform the intramammary treatment than among veterinarians without training and treating fewer cases, respectively.

#### 3.5.3. Preparing for and Administrating Treatments

More female veterinarians than male veterinarians thought that hand washing should be a part of a good DCT routine. Veterinarians treating 1–3 mastitis cases per month more often recommended hand washing than those treating more than eight cases per month. More veterinarians that had graduated in other European countries thought teat wiping with paper should be a part of the DCT routine than veterinarians that had graduated in the Nordic countries. More veterinarians without post-graduate training stated that using full insertion of the tip of the tube should be a part of the treatment routine than those with training.

#### 3.5.4. Legislation

Fewer veterinarians without post-graduate training knew about the legislation on DCT than among veterinarians with such training. The stated compliance with the legislation was associated with year of degree, region, and gender. For example, it was more common among veterinarians graduating before 2001 compared to those graduating after 2009, among veterinarians working in South Sweden compared to those working in Småland and the islands, and among female compared to male veterinarians, to state that they always/almost always complied with the legislation. The compliance with legislation also varied with number of years in cattle practice and number of mastitis cases per month. For example, the compliance was lower among veterinarians with <5 years in cattle practice than among those with 15–19 or ≥25 years in practice, and lower among veterinarians treating 9–15 cases per month than among those treating fewer cases.

#### 3.5.5. Assessment of Treatment Effects

The year of degree was associated with how often the veterinarian recommended follow-up of the effects of DCT by checking CSCC at the first milk recording after calving. For example, this was more often recommended by veterinarians graduating before 2002 than by those graduating 2015–2020. Likewise, more veterinarians with ≥25 years in practice often recommended this practice than those with <5 years in practice. The same two variables were also associated with how often the veterinarian recommended follow-up of the DCT effects by CMT after calving. For example, this recommendation was more common among veterinarians graduating before 2015 than among those graduating 2015–2020, and more common among those with ≥5 years than among those with fewer years in practice. Those two variables, along with post-graduate training, were also associated with how often the veterinarians recommended follow-up using bacteriological examination of milk samples after calving. Such recommendation was, for example, more common among veterinarians graduating before 2002 than among those graduating after 2008, and among veterinarians with ≥25 years in practice than among those with less than 10 years. This recommendation was also more common among veterinarians with post-graduate training in cattle diseases than among those without such training.

### 3.6. Questions about ITS to Farmers and Associations between Herd Variables and Answers Given by Farmers

Descriptive statistics on routines for ITS used by the farmers are given in Table 5. In short, most farmers did not use ITS. The most common reason given for this was that they did not think it was necessary due to good udder health (37%). Almost 30% stated that they did not know about ITS. Among those using ITS, the most common reasons given were recommendation from advisor (55%) or udder health problems in the herd (47%). In those herds, 56% treated all cows, mostly based on recommendation from advisor, while remaining herds treated the occasional cow (42% treated every second cow). Most of the herds using ITS did not take milk samples for bacteriology before decision on treatment. Approximately half of the herds using ITS did not think the treatment involved any risks or difficulties while 37% mentioned the risk to introduce dirt/bacteria. Among herds using ITS 46% answered that they sometimes combine ITS with DCT while 25% said that they always do so. To evaluate the effects of ITS treatment by checking the CSCC at first milk recording was common in 54% of the herds and rather common in 14% of the herds while using CMT after calving was common or rather common in 33% and 11% of the herds, respectively. It was rare to take milk samples for bacteriology after calving. Most (85%) of the farmers using ITS thought that treatment improves cow udder health in the coming lactation while 61% and 66% stated that the milk production and cow longevity, respectively, improves (20–30% answered that they did not know). Just under half (43%) of the farmers did not know if calf health was affected by ITS while 31% did not think it had any effect.

As can be seen in Table 5, five herd variables, but especially milking system, were associated with the responses given by the farmers. Detailed information on the results is given in Supplementary Table S3. Here, only a short summary with some examples is given. The use of ITS varied between regions and was, for example, more common in herds in North Middle Sweden and South Sweden than in Norrland. It was also more common to use ITS in herds producing >11,000 kg ECM than in herds with lower production, and in herds with AMS, milking parlour, or milking rotary than in herds with tie-stall milking. In herds with BMSCC <200,000 cells/mL it was more common than in herds with higher BMSCC to give the reason that the udder health was so good that ITS was not needed. It was also more common to give this explanation in herds with tie-stall milking or milking parlour than in herds with AMS, and in herds with <138 cows than in herds with more cows.

### 3.7. Questions about ITS to Veterinarians and Associations between Veterinary Variables and Answers Given by Veterinarians

Descriptive statistics on advice given on the use of ITS by veterinarians are given in Table 6. In short, almost half of the veterinarians never prescribed ITS. Among those who did, less than 15% stated that they always/rather often use bacteriology before such prescription. If prescribed, most (77%) veterinarians sent the milk samples to an accredited laboratory. It was most common to prescribe ITS to the occasional cow in the herds but approximately one-third said they prescribed ITS to all cows in a herd. The most common reason for this was herd problems with Gram-negative IMI. When selecting cows for ITS, the variable mostly used (69%) was the UHC at the last milk recording before DO. Other variables used for selection were having no case of clinical mastitis (42%), the CSCC at last milk recording (38%), and no CMT reaction at drying off (42%). Almost all veterinarians prescribing ITS recommended treatment of all four udder quarters and 85% of those veterinarians gave advice on how to perform the treatment. Those who did not give such advice stated that they did not perceive any demand for this. Among the veterinarians, 92% thought that clean gloves were important, 87% recommended wiping the teat end with provided serviette, 73% to wash hands before treatment, and 60% to wipe the teats with a moist single-use towel. Twelve percent recommended the use of full insertion while 63% recommended partial insertion of the tip of the tube into the teat canal. Almost all (93%) stated that there are risks or difficulties with treatment, most commonly lack in hygiene/risk of introducing bacteria into the udder. Most veterinarians recommended to sometimes (60%) or always (10%) combine ITS with DCT. To evaluate the effects of ITS treatment by CMT after calving was always to rather often recommended by 60% of the veterinarians while the corresponding numbers for CSCC at first milk recording was 55% and taking milk sample for bacteriology 11%. Half (52%) of the veterinarians thought that ITS treatment improves cow udder health in coming lactation while 28% thought that the milk production improves, 35% that the cow longevity improves, and 9% that calf health improves. Approximately half of the veterinarians stated that they did not know if ITS treatment had any effect on udder health (43%), calf health (56%), milk production (54%), or cow longevity (54%).

As can be seen in Table 6, three veterinary variables, but especially post-graduate training, were associated with the responses given by the veterinarians. Detailed information on the results is given in Supplementary Table S4. Here, a summary of the results with some examples is given. The likelihood that the veterinarians prescribed ITS varied between regions and was, for example, higher in Småland and the islands or West Sweden than in East Sweden. Prescribing ITS was more common among veterinarians with post-graduate training and veterinarians treating 9–15 mastitis cases per month than among veterinarians without training or treating fewer cases, respectively. Veterinarians with post-graduate training gave advice about the routines at ITS treatment more often than veterinarians without training. It was also more common that veterinarians with such training stated that ITS treatment improves udder health after calving compared to veterinarians without training who often stated that they did not know if ITS affects udder health. The same attitude was observed for veterinarians treating >1 mastitis case/month compared with those treating <1 case/month.

## 4. Discussion

This study is the first on farmer routines for DCT and ITS treatment of dairy cows in Swedish herds. Studies, covering some of the questions raised in this study, have previously been performed in Finland and Germany [13,15]. In addition, other studies have reported on proportions of cows treated in different countries, e.g., [16,17,18,19]. To our knowledge, however, this is the most comprehensive study of farmer routines and the first to investigate attitudes of the farmers to the effects of DCT and ITS, and the first to study advice and attitudes of field veterinarians on DCT and ITS treatment of dairy cows.

### 4.1. Use of DCT and Comparisons with National and International Recommendations

The results clearly showed that farmers and veterinarians followed the national recommendations to use SDCT. The results at herd level were similar to those in a recent Finnish study [13]. In contrast, most herds in Canada, Germany, and US used BDCT [15,16,17,19]. The Nordic model has always been to use SDCT to minimize prophylactic use of antibiotics [2], and this is reflected, for example, in the recommendations and guidelines in Sweden [3] and Denmark [8]. In many other countries, BDCT has been the norm for many years, but in recent years, more countries have introduced SDCT with good results reviewed by [20]. According to a recent meta-analysis the results varied between studies comparing SDCT and BDCT, but the combined data indicated a slightly higher risk for IMI after calving when using SDCT [20]. However, as the number of studies that did not use ITS in combination with DCT was small, it is difficult to distinguish the effect of the use of DCT alone from that of the combined use. In a recent Finnish study [21], significant differences in SCC and milk production were not found at herd level between herds using SDCT and herds using BDCT or no DCT.

When using SDCT it is important to select cows that are likely to benefit from the treatment to avoid unnecessary use of antibiotics. In the present study, the most common variable used by farmers and veterinarians for selection of cows was the UHC at the last milk recording before drying-off, which accords with national recommendations. However, only two-thirds of the farmers stated that they used this variable, indicating a need for improvement. Good knowledge about types of IMI and their antimicrobial susceptibility is also important when selecting cows for DCT to ensure selection of a suitable DCT product. In the present study, however, only a third of the veterinarians recommended milk sampling for bacteriology before DCT. Moreover, the results indicated that this is seldom done by the farmers, so improvements are also needed in this area. To use some measure of the CSCC to identify cows likely to have an IMI when selecting cows for DCT is also recommended by branch organisations in other countries, but the CSCC cut-offs used, and the number of milk recordings included vary [7,8]. They also state that it is important to know the pathogens present in the herd and their antimicrobial susceptibility. Several studies have been performed on how to select cows for DCT; however, despite this, the optimal selection method cannot be considered scientifically proven. Examples of tools suggested for identification of infected/non-infected cows at drying-off are: checking the CSCC at the last 1–3 milk recordings before drying off; whether or not the cow has had clinical mastitis during the lactation; and the performing of bacteriological examination of milk samples [10,20,22,23]. Other studies indicate that CMT can be used to identify udder quarters with subclinical IMI at drying-off [24,25,26]. The most common CSCC cut-off used for infected/non-infected is 200,000 cells/mL, but in other studies the cut-off has varied between 100,000 and 300,000 cells/mL [20]. As a high CSCC at the last milk recording before drying-off can indicate poor prognosis of DCT cure, the recommendation may be to cull such cows instead of using DCT [22].

According to the national recommendations, thorough cleaning of the teat end before DCT is important, preferably with alcohol, but more details were not given. However, almost none of the farmers and only a small proportion of the veterinarians stated that they use/recommend cleaning with cotton moistened with alcohol. Instead, most used, or recommended, cleaning with the provided serviette. Teat dipping or spraying directly after treatment, and control of the udder and teat dipping or spraying morning and evening for 24 h, were also included in the recommendations. These recommendations were, however, not given as alternatives in the questionnaire and only a few and none, respectively, added this routine under comments. It was clear that the description of hygienic measures when using DCT in the national recommendations was not detailed enough and needs updating. In comparison, the UK recommendations provide much more information using both pictures and videos [7].

Compliance with the recommendation to evaluate effects of DCT after calving varied markedly within both groups and the results indicate room for improvement. For example, only 60% of the farmers stated that they always examined the CSCC and only one-fourth used CMT after calving. The proportion of veterinarians that always/almost always recommended evaluation of the effects of DCT after calving was also low. Moreover, around two-thirds of the farmers did not think there were any risks or difficulties associated with DCT while the veterinarians were of the opposite opinion. The fact that so few farmers knew about the risks is especially noteworthy as the farmers are those performing the actual treatments. It was also clear that many of the veterinarians needed to improve their knowledge on, and compliance with, the national legislation on use of DCT.

At the time of the study, advice on pros and cons with full or partial insertion of the tip of the intramammary tube into the teat canal or on massage of the udder quarter after treatment was not included in the recommendations. Although this information was not provided by the pharmaceutical company responsible for the DCT products at the time of the study, their recommendation was to use partial insertion and massage the teat and udder after infusion (Manske T., personal communication, 2020). To use partial insertion of the tip of the tube rather than full insertion is also recommended in several other countries [5,6,7]. Few studies comparing partial and full insertion have, however, been published. In one study, partial insertion led to fewer new IMI and increased treatment efficacy, and in another study an association was found between being a herd with a low BMSCC and herd use of partial insertion [27,28]. The theory is that the risk for mechanical damages in the teat canal and the risk to introduce unwanted bacteria into the udder decreases when using partial insertion/short tip. It is also considered favourable that the teat canal is exposed to antibiotics as bacteria can colonize/infect the teat canal.

### 4.2. Use of ITS and Comparisons with National and International Recommendations

In the present study, less than one-fifth of the farmers used ITS and it was not so common that the veterinarians prescribed ITS. At the time of the study advice on ITS was not included in the national recommendations or guidelines. The results were somewhat lower than those reported from Finland and Germany where around one-third of the farmers reported the use of ITS [13,15]. In contrast, most farmers used ITS in UK [18], which was in line with the UK recommendations [7]. The use of ITS is also recommended and common in North America [5,6,17]. Several studies on ITS have been performed in other countries, and a recent meta-analysis indicated that ITS reduces the occurrence of new IMI at calving and the presence of clinical mastitis; although, the results vary between studies, especially if the herd has problems with environmental bacteria or not [12]. In Sweden, the main mastitis-associated pathogens are contagious bacteria making the interest of ITS smaller. Moreover, a couple of Swedish case studies did not indicate any positive effects of ITS on udder health [29,30].

According to the questionnaires, ITS was only used or recommended by a relatively small proportion of the farmers and veterinarians. Thus, the results of the questionnaires should be interpreted with care as they are based on small numbers of respondents. Almost half of the farmers using ITS were, however, aware of the risks and difficulties with ITS treatment, and the proportion was numerically higher than for the same question regarding DCT. Although the use of ITS was low, there is still the need to include ITS treatment in the national recommendations in the future.

### 4.3. Attitudes to the Importance of DCT and ITS Treatment

The attitudes to the importance of DCT for cow udder health and milk production in the beginning of the next lactation, cow longevity, and calf health varied among farmers and veterinarians. The variation within group was smallest for cow udder health and most of the respondents thought that it would improve due to DCT. Substantial proportions of farmers and veterinarians were, however, uncertain on the effects of DCT on cow longevity and calf health. Associations between DCT and better udder health have been clearly shown in several studies, e.g., [1,20,31]. It is also clear that cows with healthy udders produce more milk than cows with IMI and mastitis. Cows with healthy udders also have a lower risk for pre-mature culling improving the longevity of the cows [32]. However, whether there is an effect of DCT on calf health is less clear. It is likely that other factors are more important for calf health, but studies have shown that the absorption of colostral antibodies is reduced if the colostrum contains high numbers of bacteria [33]. This may in turn increase the risk for calf diarrhoea and reduced growth.

The attitudes to the importance of ITS for cow health and production, or for calf health, did also vary markedly within the groups. Overall, the uncertainty was rather high among the veterinarians, probably reflecting their limited experience of ITS. Among farmers responding to these questions, most thought that ITS improves cow udder health, milk production, and longevity. However, as the number of farmers using ITS was low the results must be interpreted with care. According to studies from other countries the use of ITS is associated with improved udder health and thus with better milk production and a lower risk for culling [12]. The Swedish experiences are very limited but have so far been less favourable [29,30].

### 4.4. Associations with Demographic Variables

Among the herd variables, milking system and milk production were significantly associated with the largest number of questions on DCT and ITS while production form was associated with the lowest number of questions. Among the veterinary variables, post-graduate training and number of mastitis cases per month were significantly associated with the largest number of questions, and year of degree and gender were associated with the lowest number of questions. In both groups, more associations were found between the variables and the questions on DCT than between the variables and the questions on ITS, probably reflecting the smaller number of respondents for the questions on ITS. Several of the herd variables were most likely influenced by each other; for example, where both milking system and milk production are associated with the number of cows in the herd, it is difficult to evaluate if a single variable or a combination of variables is important. Moreover, the number of cows per herd varies between regions of the country [34]. In the present study, it was not possible to perform multivariable analyses to further elucidate the effects.

All three variables, i.e., milking system, milk production, and number of cows per herd, were, for example, associated with, if, or how often DCT was used. Overall, use of DCT was more common in herds with relatively higher milk production and number of cows per herd. In addition, it was more common in AMS than in herds with tie-stall milking. These findings are in line with national data from the milk recording scheme showing that herds with more cows and herds with AMS have higher bulk milk SCC than smaller herds and other milking systems, respectively [35].

As expected, the BMSCC was associated with several questions on both DCT and ITS. The answers were in line with the larger need for DCT in herds with higher BMSCC as those have more cows with subclinical mastitis. Interestingly, herds with low BMSCC evaluated the effect of DCT directly after calving, possibly indicating higher awareness of the benefits of preventive measures.

Few differences were observed between organic and conventional herds. However, more organic farms thought there were risks or difficulties with DCT. The reasons for this finding are not clear but it is possible that organic herds are more concerned by withdrawal times and antimicrobial resistance.

For both farmers and veterinarians, region was associated with some of the questions. For example, the choice of DCT product varied between regions in both groups. For the veterinarians, differences between regions were also observed, for example, for how often they prescribe DCT and ITS. The reasons for these differences are not known but aspects like tradition among farmers and veterinarians may be of importance. The through-put of veterinarians may also vary between regions.

Year of degree and years in cattle practice are both indicators of experience and probably also type of education and tradition. It was therefore not surprising that the variables had similar associations with factors such as compliance with legislation and evaluation of the effects of DCT. Other factors indicating differences in experience but also in interest in cattle diseases were post-graduate training and number of mastitis cases/month. It was not surprising that both variables were associated with the answers to several questions and that the answers indicated better knowledge about DCT and ITS, and better compliance with recommendations among those with post-graduate training and many cases per month.

### 4.5. Methodological Considerations

Unfortunately, the proportions of respondents were rather low for both farmers (14%) and veterinarians (25%). However, a similar response rate (13%) was also observed in a web-based study on farmers in Finland [13] while a web-based study on mastitis sent to Swedish veterinarians had a higher response rate (36%) [36]. The reasons for the low numbers of respondents are not known but lack of time is probably an important factor. It is also possible that a web-based questionnaire results in lower response rate than other types of questionnaires. For example, Bertulat et al. [15] had a response rate of 49% for a questionnaire to milk producers performed in connection with a physical meeting and McDougall et al. [28] had a response rate of 44% when sending the questionnaire via postal mail.

Questionnaire studies must always be interpreted with care as respondents may not be representative for the population in question. In our study, the farmers participating were well spread geographically in the country but the proportion of herds with free-stalls and with AMS as well as the number of cows per herd was larger than the average among herds affiliated to the official cow control scheme for that year (55%, 33%, and 92 cows, respectively) [34]. Moreover, the results indicate that participating herds also had higher milk production and lower BMSCC than the average herd (10,417 kg ECM/cow and 211,000 cells/mL, respectively) (Nyman, A.-K., personal communication 2021). Unfortunately, we were not able to control if the responding veterinarians were representative for the target group. However, in a previous web-based questionnaire study to veterinarians [36], the distribution of gender and year of veterinary degree among respondents did not differ from the target group. Given the facts mentioned above on representativity in combination with the relatively low response rate, the results, including proportions of cows treated with DCT and/or ITS, must be interpreted with care.

The results should also be interpreted with caution as the risk for type I errors, to find significant results even though there are no true associations, increases when many risk factors are tested. Hence, some of the associations found might be just due to chance. As it is impossible to know which of these associations could be due to chance, additional studies are needed to further confirm the findings in this study.

## 5. Conclusions

The routines used by the farmers and the advice given by the veterinarians responding to the questionnaires were in many areas in line with the recommendations available at the time, but the answers also indicated room for improvement in some areas. We also found interesting associations between routines used and advice given and the tested herd and veterinary variables, respectively. The results, as well as those on attitudes to the effects of DCT and ITS on animal health and production, indicated a need for more education. We also found that the existing recommendations were insufficient and in need of an up-date. Therefore, new national recommendations were produced and spread among target groups.

## Figures and Tables

**Table 1:** Distribution of herds (*n* = 340) participating in a web-based questionnaire on routines for drying-off and dry period among herd variables.

Variables	Categories	Herd *n* (%)
Region	Norrland	49 (14)
	Northern Middle Sweden	30 (9)
	East Sweden	51 (15)
	Småland and the islands	72 (21)
	South Sweden	44 (13)
	West Sweden	92 (27)
Production type	Conventional	271 (80)
	Organic	67 (20)
Housing system	Free-stalls, insulated	168 (50)
	Free-stalls, not insulated	67 (20)
	Tie-stalls, short	79 (23)
	Tie-stalls, long	21 (6)
	Uncertain	2 (1)
Milking system	Automatic milking system	142 (42)
	Tied-up	105 (31)
	Parlour	79 (23)
	Rotary	9 (3)
	Combinations	2 (1)
Number of cows (mean (SD) = 116 (125))	<53	84 (25)
	53–77	83 (25)
	78–137	85 (25)
	≥138	85 (25)
Milk production, kg ECM/cow/year	<9000	40 (12)
	9000–11,000	195 (58)
	>11,000	101 (30)
Bulk milk somatic cell count, cells/mL	<200,000	222 (66)
	200,000–300,000	110 (33)
	>300,000	4 (1)

**Table 2:** Distribution of veterinarians (*n* = 130) participating in a web-based questionnaire on advice on drying-off and dry period among veterinary variables.

Variables	Categories	Veterinarians *n* (%)
Region	Norrland	23 (18)
	Northern Middle Sweden	18 (14)
	East Sweden	33 (25)
	Småland and the islands	18 (14)
	South Sweden	15 (11)
	West Sweden	23 (18)
Year of veterinary degree	1977–1991	26 (20)
	1992–2001	26 (20)
	2002–2008	24 (18)
	2009–2014	25 (19)
	2015–2020	29 (22)
Country of veterinary degree	Sweden	105 (81)
	Denmark/Finland/Norway	14 (11)
	Other European countries	11 (8)
Gender	Female	101 (78)
	Male	28 (21)
	Do not want to answer	1 (1)
Post-graduate training	Yes	77 (60)
	No	52 (40)
Years in cattle practice	<5	30 (23)
	5–9	22 (17)
	10–14	19 (15)
	15–19	21 (16)
	20–24	18 (14)
	≥25	20 (15)
Number of mastitis cases/month	<1	17 (13)
	1–3	20 (23)
	4–8	40 (31)
	9–15	27 (21)
	>15	16 (12)

**Table 3:** Responses to questions about routines regarding dry-cow therapy (DCT) of dairy cows with intramammaries containing antibiotics given by farmers (*n* = 340) participating in a web-based questionnaire. The associations between herd variables and the answers were investigated using univariable regression models and if significant (*p* ≤ 0.05) the herd variables are presented.

Questions	Categories	Herd *n* (%)	Significant Herd Variables ^1^
Do you use DCT?			MP, NC
	Yes	284 (84)	
	No	54 (16)	
Why do you use DCT (if yes)?			
	Udder health problems in herd	143 (51)	BMSCC
	Recommended by advisor	106 (37)	MP
	Other	71 (25)	
Did you treat some or all cows last year?			ns
	Some	273 (96)	
	All	11 (4)	
Did you take milk samples for bacteriology before DCT decision?			ns
	Yes, from all cows	26 (10)	
	Yes, from some cows	113 (41)	
	No	134 (49)	
How many cows got DCT last year (if some cows)?			BMSCC, MP, MS, NC
	Approximately three of four	2 (1)	
	Approximately every second	32 (12)	
	Approximately one of four	104 (38)	
	The occasional cow	135 (49)	
Which factor/s affected the choice of cows for DCT (if some cows)?			ns
	If the cow had clinical mastitis during lactation	123 (45)	
	The cow SCC at last test milking before drying-off	134 (49)	
	The cows UDS at last test milking before drying-off	175 (64)	
	Positive CMT at drying-off	80 (29)	
	Bacteriological result milk sample	69 (25)	
Which intramammaries ^2^ did you use last year (if some cows)?			
	Carepen^®^	34 (13)	
	Benestermycin^®^	102 (38)	MP, R
	Siccalactin^®^	204 (75)	MP, R
Do you always treat all four udder quarters?			ns
	Yes	228 (84)	
	No	45 (16)	
Do you always use the same routine when performing DCT (if some)?			na
	Yes	268 (99)	
	No	4 (1)	
Which of the following is/are usually done at DCT (if some)?			
	Wash hands before DCT	140 (51)	MS
	Use of clean gloves	110 (40)	MP, MS
	Wiping teats with paper	70 (26)	PT
	Wiping teats with moist single-use cloth	97 (36)	MS
	Wiping teat end with provided serviette	251 (92)	na
	Wiping teat end with cotton moistened with alcohol	10 (4)	na
	Use of long tip (full insertion)	225 (82)	R
	Use of short tip (partial insertion)	26 (10)	na
	Massaging the teat/udder quarter after infusion of product	100 (37)	ns
	Other	13 (5)	na
Are there any risks or difficulties with the DCT (if some)?			PT
	Yes	78 (29)	
	No	194 (71)	
How often do you evaluate the DCT effect by checking SCC at first test milking?			NC
	Always/almost always	161/58)	
	Quite often	52 (19)	
	Less often	24 (9)	
	Never/almost never	40 (14)	
How often do you evaluate the DCT effect by checking CMT after calving?			BMSCC, MS
	Always/almost always	71 (27)	
	Quite often	47 (18)	
	Less often	59 (22)	
	Never/almost never	87 (33)	
How often do you evaluate the DCT effect by taking milk samples for bacteriology after calving?			ns
	Always/almost always	6 (2)	
	Quite often	13 (5)	
	Less often	66 (26)	
	Never/almost never	167 (66)	
How do you think DCT affects the following aspects of animal health and production during early lactation?			na
	Cow udder health		
	Improves	259 (92)	
	No effect	4 (1)	
	Worsens	0 (0)	
	Don’t know	20 (7)	
	Calf health		
	Improves	22 (8)	
	No effect	97 (35)	
	Worsens	3 (1)	
	Don’t know	154 (56)	
	Milk production		
	Improves	178 (64)	
	No effect	33 (12)	
	Worsens	0 (0)	
	Don’t know	69 (25)	
	Cow longevity		
	Improves	176 (63)	
	No effect	20 (7)	
	Worsens	2 (1)	
	Don’t know	82 (29)	

^1^ ns = not significant; na = not analysed due to too low variation in answers or that the relevance of investigating associations was deemed low; BMSCC = bulk milk somatic count; MP = milk production; MS = milking system; NC = number of cows/herd; PT = production type; R = region. ^2^ For explanation of intramammary products see material and methods.

**Table 4:** Responses to questions about dry-cow therapy (DCT) of dairy cows with intramammaries containing antibiotics given by veterinarians (*n* = 130) participating in a web-based questionnaire. The associations between veterinary variables and the answers were investigated using univariable regression models and if significant (*p* ≤ 0.05) the veterinary variables are presented.

Questions	Categories	Herd *n* (%)	Significant Veterinary Variables ^1^
How often do you prescribe intramammaries ^2^ for DCT?			MC, PT, R
	Every week	20 (15)	
	Some time each month	64 (49)	
	Less than some time each month	42 (32)	
	Never	4 (3)	
Do you use bacteriological diagnostics before prescribing DCT?			ns
	Always/almost always	20 (16)	
	Quite often	24 (19)	
	Less often	53 (42)	
	Never/almost never	29 (23)	
Where do you perform the bacteriology?			
	Own culture	52 (54)	R
	At accredited laboratory	68 (70)	MC
	Both own culture and accredited laboratory	24 (25)	
Do you usually prescribe the same kind of intramammaries?			na
	Yes	114 (90)	
	No	12 (10)	
If Yes, which intramammaries do you prescribe?			
	Benestermycin^®^	35 (30)	MC, PT, R
	Siccalactin^®^	78 (68)	MC, PT, R
	Both Benestermycin^®^ and Siccalactin^®^	2 (2)	
How often do you recommend DCT to all or some cows?			
All			na
	Always/almost always	0 (0)	
	Quite often	1 (1)	
	Less often	9 (7)	
	Never/almost never	110 (92)	
Some			PT
	Always/almost always	58 (46)	
	Quite often	52 (41)	
	Less often	16 (13)	
	Never/almost never	0 (0)	
Which of the following factor/s affects your choice of cows for DCT?			
	If the cow had clinical mastitis during lactation	71 (58)	ns
	The cow SCC at the last test milking before drying-off	51 (41)	ns
	The cows UDS at the last test milking before drying-off	108 (88)	ns
	Positive CMT at drying-off	50 (41)	PT
	Bacteriological result milk sample	78 (63)	ns
	Other	0 (0)	na
Do you always recommend treatment of all four udder quarters?			na
	Yes	113 (90)	
	No	13 (10)	
Do you give advice on how to perform the actual treatment?			MC, PT
	Yes, often	12 (10)	
	Yes, sometimes	61 (51)	
	No	46 (39)	
Why do you not give such advice?			na
	Do not have enough knowledge	15 (33)	
	Lack of time	3 (7)	
	No demand	38 (83)	
	Other	8 (17)	
Which of the following do you think should be a part of a good routine at DCT (if give advice)?			
	Wash hands before DCT	56 (72)	G, MC
	Use of clean gloves	70 (90)	ns
	Wipe teats with paper	20 (26)	CD
	Wipe teats with moist single-use cloth	52 (67)	ns
	Wipe teat ends with provided serviett	69 (88)	ns
	Wipe teat ends with cotton moistened with alcohol	16 (21)	ns
	Use of long tip (full insertion)	35 (45)	PT
	Use of short tip (partial insertion)	22 (28)	ns
	Massage the teat/udder quarter after infusion of product	47 (60)	ns
	Other	14 (18)	na
Are there any risks or difficulties with DCT?			na
	Yes, poor hygiene	61 (48)	
	Yes, antibiotic resistance	29 (24)	
	Yes, withdrawal time	14 (12)	
	Yes, farmer trauma	4 (3)	
	Yes, teat trauma	12 (10)	
	No	14 (11)	
	Don’t know	4 (3)	
Do you know about the national legislation on DCT?			PT
	Yes	87 (67)	
	No	42 (33)	
How often do you follow the legislation on DCT?			G, MC, R, YD
	Always/almost always	63 (50)	
	Quite often	42 (33)	
	Less often	18 (14)	
	Never/almost never	3 (2)	
How often do you recommend follow-up of the DCT by checking SCC at first test milking?			YD, YP
	Always/almost always	44 (36)	
	Quite often	38 (31)	
	Less often	15 (12)	
	Never/almost never	24 (20)	
How often do you recommend follow-up of the DCT by checking CMT after calving?			YD, YP
	Always/almost always	41 (34)	
	Quite often	38 (31)	
	Less often	25 (20)	
	Never/almost never	18 (15)	
How often do you recommend follow-up of the DCT by bacteriology after calving?			PT, YD, YP
	Always/almost always	5 (4)	
	Quite often	11 (10)	
	Less often	51 (44)	
	Never/almost never	48 (42)	
How do you think DCT affects the following aspects of animal health and production during early lactation?			
			na
	Cow udder health	112 (86)	
	Improves	0 (0)	
	No effect	0 (0)	
	Worsens	18 (14)	
	Don’t know		ns
	Calf health	18 (14)	
	Improves	52 (41)	
	No effect	1 (1)	
	Worsens	57 (44)	
	Don’t know		ns
	Milk production	77 (60)	
	Improves	18 (14)	
	No effect	0 (0)	
	Worsens	34 (26)	
	Don’t know		na
	Cow longevity	85 (65)	
	Improves	9 (7)	
	No effect	0 (0)	
	Worsens	36 (28)	
	Don’t know		

^1^ ns = not significant; na = not analysed due to too low variation in answers or that the relevance of investigating associations was deemed low; G = gender; CD = country of degree; MC = number of mastitis cases/month; PT = post-graduate training; YP = number of years in cattle practice; YD = year of degree; R = region. ^2^ For explanation of intramammary products see material and methods.

**Table 5:** Responses to questions about routines regarding treatment of dairy cows with internal teat sealants (ITS) at drying-off given by farmers (*n* = 340) participating in a web-based questionnaire. The associations between herd variables and the answers were investigated using univariable regression models and if significant (*p* ≤ 0.05) variables are presented.

Questions	Categories	Herd *n* (%)	Significant Herd Variables ^1^
Do you use ITS?			MP, MS, R
	Yes	61 (18)	
	No	277 (82)	
Why do you not use ITS (if No)?			
	Cows have so good udder health that it is not needed	100 (37)	BMSCC, MS, NC
	Treatment is too expensive	27 (10)	na
	Treatment is too time-consuming	35 (13)	na
	Other, did not know about ITS	71 (26)	na
	Other, have not heard any good about ITS	24 (9)	na
Why did you use ITS (if Yes)?			na
	Due to udder health problems	28 (47)	
	Recommended by advisor	33 (55)	
	Other	17 (28)	
Did you treat some or all cows during the last year (if Yes)?			na
	Some	26 (44)	
	All	33 (56)	
Do you use ITS in combination with dry cow therapy with antibiotics (if Yes)?			ns
	Yes, always	15 (25)	
	Yes, sometimes	28 (46)	
	No	18 (29)	
How do you think ITS affects the following aspects of animal health and production in early lactation?			na
	Cow udder health		
	Improves	50 (85)	
	No effect	3 (5)	
	Worsens	0 (0)	
	Don’t know	6 (10)	
	Calf health		
	Improves	15 (26)	
	No effect	18 (31)	
	Worsens	0 (0)	
	Don’t know	25 (43)	
	Milk production		
	Improves	36 (61)	
	No effect	12 (20)	
	Worsens	0 (0)	
	Don’t know	11 (19)	
	Cow longevity		
	Improves	39 (66)	
	No effect	3 (5)	
	Worsens	0 (0)	
	Don’t know	17 (29)	

^1^ ns = not significant; na = not analysed due to too low variation in answers or that the relevance of investigating associations was deemed low; BMSCC = bulk milk somatic count; MP = milk production; MS = milking system; NC = number of cows/herd; PT = production type; R = region.

**Table 6:** Responses to questions about advice regarding treatment of dairy cows with internal teat sealants (ITS) at drying-off given by veterinarians (*n* = 130) participating in a web-based questionnaire. The associations between veterinary variables and the answers were investigated using univariable regression models and if significant (*p* ≤ 0.05) the veterinary variables are presented.

Questions	Categories	Herd *n* (%)	Significant Veterinary Variables ^1^
How often do you prescribe ITS?			MC, PT, R
	Every week	3 (2)	
	Some time each month	32 (25)	
	More rarely than some time each week	36 (28)	
	Never	59 (45)	
Do you use bacteriological diagnostics before prescribing ITS?			ns
	Always/almost always	8 (11)	
	Quite often	11 (15)	
	Less often	22 (31)	
	Never/almost never	30 (42)	
Where do you perform the bacteriology?			na
	Own culture	19 (49)	
	At accredited laboratory	30 (77)	
	Both own culture and accredited laboratory	10 (24)	
How often do you recommend ITS to all or some cows?			na
All			
	Always/almost always	7 (10)	
	Quite often	18 (26)	
	Less often	16 (23)	
	Never/almost never	28 (41)	
Some			
	Always/almost always	6 (9)	
	Quite often	19 (28)	
	Less often	27 (40)	
	Never/almost never	16 (23)	
Do you give advice on how to perform ITS treatment?			PT
	Yes	60 (85)	
	No	11 (15)	
Do you recommend use of ITS in combination with dry cow therapy with antibiotics (if Yes)?			ns
	Yes, always	7 (10)	
	Yes, sometimes	42 (60)	
	No	21 (30)	
How do you think ITS affects the following aspects of animal health and production?			
			MC, PT
	Cow udder health		
	Improves	67 (52)	
	No effect	6 (5)	
	Worsens	1 (1)	
	Don’t know	55 (43)	ns
	Calf health		
	Improves	12 (9)	
	No effect	43 (34)	
	Worsens	1 (1)	
	Don’t know	72 (56)	ns
	Milk production		
	Improves	36 (28)	
	No effect	22 (17)	
	Worsens	1 (1)	
	Don’t know	69 (54)	ns
	Cow longevity		
	Improves	44 (35)	
	No effect	14 (10)	
	Worsens	1 (1)	
	Don’t know	69 (54)	

^1^ ns = not significant; na = not analysed due to too low variation in answers or that the relevance of investigating associations was deemed low; MC = number of mastitis cases/month; PT = post-graduate training; R = region.

## Data Availability

The datasets generated and analysed during this study are available upon reasonable request.

## Supplementary Materials

The following are available online at https://www.mdpi.com/article/10.3390/ani11123411/s1, Document S1: Questionnaire (in Swedish) presented to Swedish dairy farmers on routines for dry cow therapy with antibiotics and treatment with internal teat sealants, Document S2: Questionnaire (in Swedish) presented to Swedish cattle veterinarians on advice on dry cow therapy with antibiotics and treatment with internal teat sealants, Table S1: Significant associations between herd variables and responses to questions about routines for dry cow therapy with antibiotics of dairy cows given by farmers (*n* = 338), Table S2: Significant associations between veterinary variables and responses to questions about advice on dry cow therapy with antibiotics of dairy cows given by veterinarians (*n* = 130), Table S3: Significant associations between herd variables and responses to questions about routines for treating dairy cows with internal teat sealants at drying-off given by farmers (*n* = 338), Table S4: Significant associations between veterinary variables and responses to questions about advice on treating dairy cows with internal teat sealants at drying-off given by veterinarians (*n* = 130).
