# Supplementary material for: Herd Routines and Veterinary Advice Related to Dry-Cow Therapy and Treatment with Internal Teat Sealants in Dairy Cows
Source: Animals (Basel). 2021 Nov 29;11(12):3411. doi: 10.3390/ani11123411 (PMC8697970; doi:10.3390/ani11123411)
Supplement: Supplementary file 1 [file animals-11-03411-s001.zip › animals-1441704-supplementary.pdf]

Supplementary Document S1. Questionnaire (in Swedish) presented to Swedish dairy farmers on routines for dry cow therapy with antibiotics and treatment with internal teat sealants

Sinläggning, sintidsbehandling och sinperiod

Syftet med denna enkät är att undersöka rutiner för sinläggning, sintidsbehandling och sinperiod av/för mjölkkor på svenska gårdar. En liknande enkät skickas även till svenska veterinärer med nötkreaturspraktik. Resultaten kommer att göras tillgängliga för alla och svaren ska ligga till grund för bedömning av behov av information om dessa områden. Vi är därför mycket tacksamma för att du vill fylla i enkäten som tar cirka 10-20 minuter. Enkäten är anonym.

Projektet är ett samarbete mellan Statens veterinärmedicinska anstalt (SVA) och Växa Sverige.

Om du har några frågor kan du på dagtid nå projektledare Karin Persson Waller, SVA, via epost (karin.persson-waller@sva.se) eller telefon (018-674672).

GRUNDLÄGGANDE FRÅGOR OM DIN BESÄTTNING

1) .

2) Hur många kor (årsmedelkor) finns i besättningen?

3) I vilket län finns din besättning?

- ☐ Stockholms län
- ☐ Uppsala län
- ☐ Södermanlands län
- ☐ Östergötlands län
- ☐ Jönköpings län
- ☐ Kronobergs län
- ☐ Kalmar län
- ☐ Gotlands län
- ☐ Blekinge län
- ☐ Skåne län
- ☐ Hallands län
- ☐ Västra Götalands län
- ☐ Värmlands län
- ☐ Örebro län
- ☐ Västmanlands län
- ☐ Dalarnas län
- ☐ Gävleborgs län
- ☐ Västernorrlands län

- Jämtlands län
- Västerbottens län
- Norrbottens län

4) Vilken typ av produktion har din besättning?

- Konventionell
- Krav
- Annan ekologisk

5) Vilken är besättningens årsmedelproduktion?

- Under 9 000 kg ECM
- 9 000 till 11 000 kg ECM
- Över 11 000 kg ECM

6) Vilket är besättningens tankcelltal (årsmedeltal)?

- Under 200 000/ml
- 200 000 till 300 000/ml
- Över 300 000/ml

7) Hur mjölkas de flesta korna?

- Uppbundet
- Mjölkgrop
- Mjölkrobot
- Annat, ange vad

FRÅGOR OM SINLÄGGNINGEN

Definition: Sinläggningen är den period i slutet på laktationen då man förbereder kon innan hon går i sin dvs innan man helt slutar att mjölka kon.

9) Har ni skrivna rutiner för sinläggningen?

- Nej
- Ja

10) Hur många personer tar hand om korna under sinläggningen?

- En person
- 2-3 personer

Fler än 3 personer

11) Mjölkas korna med förlängt intervall under sinläggningen?

Nej

Ja, ibland/ganska ofta (färre än tre av fyra kor)

Ja, för det mesta (minst tre av fyra kor)

Denna informationsbox visas endast i läge förhandsgranskningen.

Följande kriterium måste vara uppfyllda för att följande fråga ska visas:

Om frågan Mjölkas korna med förlängt intervall under sinläggningen? innehåller något av dessa svarsalternativ

- Nej

12) Hur/när avslutas mjölkningen?

Mjölkningen avslutas tvärt när kon har en viss tid kvar till kalvningen (ange antal veckor i fältet nedan)

Mjölkningen avslutas tvärt när mjölkmängden är under en viss dygnsvolym (ange antal kg/dag i fältet nedan)

Annat, ange vad

Denna informationsbox visas endast i läge förhandsgranskningen.

Följande kriterium måste vara uppfyllda för att följande fråga ska visas:

Om frågan Mjölkas korna med förlängt intervall under sinläggningen? innehåller något av dessa svarsalternativ

- Nej

13)

Denna informationsbox visas endast i läge förhandsgranskningen.

Följande kriterium måste vara uppfyllda för att följande fråga ska visas:

Om frågan Mjölkas korna med förlängt intervall under sinläggningen? innehåller något av dessa svarsalternativ

- Ja, för det mesta (minst tre av fyra kor)

14) Hur ofta mjölkas korna vanligen under sinläggningen?

En gång per dag

En gång varannan dag

Annat intervall, ange vad

Denna informationsbox visas endast i läge förhandsgranskningen.

Följande kriterium måste vara uppfyllda för att följande fråga ska visas:

Om frågan Mjölkas korna med förlängt intervall under sinläggningen? innehåller något av dessa svarsalternativ

- Ja, för det mesta (minst tre av fyra kor)

15) Hur länge mjölkas korna med förlängt intervall?

- Ca 1 vecka
- Ca 2 veckor
- Annat, ange antal dagar

Denna informationsbox visas endast i läge förhandsgranskningen.

Följande kriterium måste vara uppfyllda för att följande fråga ska visas:

Om frågan Mjölkas korna med förlängt intervall under sinläggningen? innehåller något av dessa svarsalternativ

- Ja, ibland/ganska ofta (färre än tre av fyra kor)

16) Hur många av alla kor mjölkas med följande intervall under sinläggningen? (ett svar per rad)

	Inga	Färre än hälften	Cirka hälften	Fler än hälften
En gång per dag				
En gång varannan dag				
Annat intervall				

Denna informationsbox visas endast i läge förhandsgranskningen.

Följande kriterium måste vara uppfyllda för att följande fråga ska visas:

Om frågan Mjölkas korna med förlängt intervall under sinläggningen? innehåller något av dessa svarsalternativ

- Ja, ibland/ganska ofta (färre än tre av fyra kor)

17) Hur lång tid mjölkas korna med förlängt intervall under sinläggningen? (ange antal kor per rad)

	Inga	Färre än hälften	Cirka hälften	Fler än hälften
Ca 1 vecka				
Ca 2 veckor				
Annan tid				

Denna informationsbox visas endast i läge förhandsgranskningen.

Följande kriterium måste vara uppfyllda för att följande fråga ska visas:

Om frågan Mjölkas korna med förlängt intervall under sinläggningen? innehåller något av dessa svarsalternativ

- Ja, ibland/ganska ofta (färre än tre av fyra kor)

18) Varför varierar mjölkningsrutinerna mellan korna?

19) Ändras utfodringen under sinläggningen?

- Nej
- Ja, ibland/ganska ofta (färre än tre av fyra kor)
- Ja, för det mesta (minst tre av fyra kor)

Denna informationsbox visas endast i läge förhandsgranskningen.

Följande kriterium måste vara uppfyllda för att följande fråga ska visas:

Om frågan Ändras utfodringen under sinläggningen? innehåller något av dessa svarsalternativ

- Ja, ibland/ganska ofta (färre än tre av fyra kor)

20) För hur många av alla kor ändras utfodringen?

- Färre än hälften
- Cirka hälften
- Fler än hälften

Denna informationsbox visas endast i läge förhandsgranskningen.

Följande kriterium måste vara uppfyllda för att följande fråga ska visas:

Om frågan Ändras utfodringen under sinläggningen? innehåller något av dessa svarsalternativ

- Ja, ibland/ganska ofta (färre än tre av fyra kor)

21) Om utfodringen ändras - gör man lika för alla kor?

- Nej
- Ja

Denna informationsbox visas endast i läge förhandsgranskningen.

Följande kriterium måste vara uppfyllda för att följande fråga ska visas:

Om frågan Om utfodringen ändras - gör man lika för alla kor? innehåller något av dessa svarsalternativ

- Nej

22) Varför varierar utfodringen mellan korna?

Denna informationsbox visas endast i läge förhandsgranskningen.

Följande kriterium måste vara uppfyllda för att följande fråga ska visas:

Om frågan Om utfodringen ändras - gör man lika för alla kor? innehåller något av dessa svarsalternativ

- Ja

23) Hur ändras utfodringen under sinläggningen? (minst ett svar per rad)

	Minskar	Oförändrad	Ökar	Annat parti
Totala mängden kraftfoder	☐	☐	☐	☐
Totala mängden grovfoder	☐	☐	☐	☐
Mängden halm	☐	☐	☐	☐
Mängden hö	☐	☐	☐	☐
Mängden ensilage	☐	☐	☐	☐
Annat grovfoder	☐	☐	☐	☐

Denna informationsbox visas endast i läge förhandsgranskningen.

Följande kriterium måste vara uppfyllda för att följande fråga ska visas:

Om frågan Om utfodringen ändras - gör man lika för alla kor? innehåller något av dessa svarsalternativ

- Ja

24) Hur länge går korna på sinläggningsfoder?

- ☐ Ca 1 vecka
- ☐ Ca 2 veckor
- ☐ Annan tid, ange antal dagar

Denna informationsbox visas endast i läge förhandsgranskningen.

Följande kriterium måste vara uppfyllda för att följande fråga ska visas:

Om frågan Ändras utfodringen under sinläggningen? innehåller något av dessa svarsalternativ

- Ja, för det mesta (minst tre av fyra kor)

25) Hur ändras utfodringen under sinläggningen? (minst ett svar per rad)

	Minskar	Oförändrad	Ökar	Annat parti
Totala mängden kraftfoder	☐	☐	☐	☐
Totala mängden grovfoder	☐	☐	☐	☐
Mängden halm	☐	☐	☐	☐
Mängden hö	☐	☐	☐	☐
Mängden ensilage	☐	☐	☐	☐
Annat grovfoder	☐	☐	☐	☐

Denna informationsbox visas endast i läge förhandsgranskningen.

Följande kriterium måste vara uppfyllda för att följande fråga ska visas:

Om frågan Ändras utfodringen under sinläggningen? innehåller något av dessa svarsalternativ

- Ja, för det mesta (minst tre av fyra kor)

26) Hur länge går korna på sinläggningsfoder?

- [ ] Ca 1 vecka
- [ ] Ca 2 veckor
- [ ] Annan tid, ange antal dagar

27) Ändras vattentillgången under sinläggningen?

- [ ] Nej
- [ ] Ja, ibland/ganska ofta (färre än tre av fyra kor)
- [ ] Ja, för det mesta (minst tre av fyra kor)

Denna informationsbox visas endast i läge förhandsgranskningen.

Följande kriterium måste vara uppfyllda för att följande fråga ska visas:

Om frågan Ändras vattentillgången under sinläggningen? innehåller något av dessa svarsalternativ

- Ja, för det mesta (minst tre av fyra kor)

28) Hur ändras vattentillgången?

- [ ] Vattnet tas bort helt
- [ ] Mängden minskar
- [ ] Mängden ökar
- [ ] Annat, ange vad

Denna informationsbox visas endast i läge förhandsgranskningen.

Följande kriterium måste vara uppfyllda för att följande fråga ska visas:

Om frågan Ändras vattentillgången under sinläggningen? innehåller något av dessa svarsalternativ

- Ja, ibland/ganska ofta (färre än tre av fyra kor)

29) Hur många av alla kor får (ett alternativ per rad)

	Inga	Färre än hälften	Cirka hälften	Fler än hälften
Samma vattentillgång	☐	☐	☐	☐
Ökad vattentillgång	☐	☐	☐	☐
Minskad vattentillgång	☐	☐	☐	☐
Inget vatten	☐	☐	☐	☐
Annat	☐	☐	☐	☐

Denna informationsbox visas endast i läge förhandsgranskningen.

Följande kriterium måste vara uppfyllda för att följande fråga ska visas:

Om frågan Ändras vattentillgången under sinläggningen? innehåller något av dessa svarsalternativ

- Ja, ibland/ganska ofta (färre än tre av fyra kor)

30) Varför varierar vattenrutinerna mellan korna?

31) Går korna kvar i mjölkgruppen under sinläggningen?

- Nej
- Ja, ibland/ganska ofta (färre än tre av fyra kor)
- Ja, för det mesta (minst tre av fyra kor)

Denna informationsbox visas endast i läge förhandsgranskningen.

Följande kriterium måste vara uppfyllda för att följande fråga ska visas:

Om frågan Går korna kvar i mjölkgruppen under sinläggningen? innehåller något av dessa svarsalternativ

- Nej

32) Flyttas korna till en annan stallbyggnad?

- Nej
- Ja

Denna informationsbox visas endast i läge förhandsgranskningen.

Följande kriterium måste vara uppfyllda för att följande fråga ska visas:

Om frågan Går korna kvar i mjölkgruppen under sinläggningen? innehåller något av dessa svarsalternativ

- Nej

33) Är korna fortfarande inom höravstånd från mjölkningen?

- Nej
- Ja

34) Spendoppas/-sprejas de flesta korna (minst tre av fyra kor) under sinläggningen?

- Nej
- Ja, efter varje mjölkning
- Ja, vid fasta tider under dygnet
- Ja, vid annan tid ange när

35) Undersöks juvret (t ex tittar, känner, CMT-paddlar) på de flesta korna (minst tre av fyra) under sinläggningen?

- Nej
- Ja

Denna informationsbox visas endast i läge förhandsgranskningen.

Följande kriterium måste vara uppfyllda för att följande fråga ska visas:

Om frågan Undersöks juvret (t ex tittar, känner, CMT-paddlar) på de flesta korna (minst tre av fyra) under sinläggningen? innehåller något av dessa svarsalternativ

- Ja

36) Hur undersöks juvret? (flera alternativ möjliga)

- [ ] Tittar på juvret
- [ ] Känner på juvret
- [ ] CMT-paddlar
- [ ] Annat, ange vad

Denna informationsbox visas endast i läge förhandsgranskningen.

Följande kriterium måste vara uppfyllda för att följande fråga ska visas:

Om frågan Undersöks juvret (t ex tittar, känner, CMT-paddlar) på de flesta korna (minst tre av fyra) under sinläggningen? innehåller något av dessa svarsalternativ

- Ja

37) När undersöks juvret?

- Efter varje mjölkning
- Mellan mjölkningar
- Annat, ange när

38) Använder ni några andra preparat (utom spendopp/-sprej, sintidsantibiotika eller spenförslutare) under sinläggningen?

- Nej
- Ja, homeopatiska preparat
- Ja, annat, ange vad och hur

Denna informationsbox visas endast i läge förhandsgranskningen.

Följande kriterium måste vara uppfyllda för att följande fråga ska visas:

Om frågan Använder ni några andra preparat (utom spendopp/-sprej, sintidsantibiotika eller spenförslutare) under sinläggningen? innehåller något av dessa svarsalternativ

- Ja, homeopatiska preparat

39) Till hur många av korna används homeopatiska preparat?

Färre än hälften

Cirka hälften

Fler än hälften

40) Anser du att sinläggningsrutinerna är viktiga för följande aspekter på kornas hälsa och produktion under början av kommande laktation? (ange ett alternativ per rad)

	Nej	Ja	Vet ej
Kons juverhälsa			
Kons mjölkproduktion			
Kons fruktsamhet			

FRÅGOR OM SINTIDSBEHANDLING MED ANTIBIOTIKA

Definition: Sintidsbehandling med antibiotika innebär att antibiotika (oftast långtidsverkande) sprutas in i alla juverdelar via spenkanalen efter sista mjölkningen under sinläggningen dvs precis innan sinperioden tar vid.

42) Används sintidsbehandling med antibiotika i besättningen?

Nej

Ja

Denna informationsbox visas endast i läge förhandsgranskningen.

Följande kriterium måste vara uppfyllda för att följande fråga ska visas:

Om frågan Används sintidsbehandling med antibiotika i besättningen? innehåller något av dessa svarsalternativ

- Nej

43) Varför används inte sådan sintidsbehandling? (flera alternativ möjliga)

- Korna har så god juverhälsa att det inte behövs
- Behandlingen är för dyr
- Behandlingen är för arbetskrävande
- Oro för antibiotikaresistens
- Annat, ange vad

Denna informationsbox visas endast i läge förhandsgranskningen.

Följande kriterium måste vara uppfyllda för att följande fråga ska visas:

Om frågan Används sintidsbehandling med antibiotika i besättningen? innehåller något av dessa svarsalternativ

- Ja

44) Varför används sådan sintidsbehandling? (flera alternativ möjliga)

- På grund av juverhälsoproblem i besättning

☐ Rekommenderas av rådgivare
☐ Annat, ange vad

Denna informationsbox visas endast i läge förhandsgranskningen.

Följande kriterium måste vara uppfyllda för att följande fråga ska visas:

Om frågan Används sintidsbehandling med antibiotika i besättningen? innehåller något av dessa svarsalternativ

- Ja

45) Sintidsbehandlades vissa eller alla kor under senaste året?

- Vissa kor
- Alla kor

Denna informationsbox visas endast i läge förhandsgranskningen.

Följande kriterium måste vara uppfyllda för att följande fråga ska visas:

Om frågan Sintidsbehandlades vissa eller alla kor under senaste året? innehåller något av dessa svarsalternativ

- Vissa kor

46) Tog ni mjölkprover för bakteriologisk undersökning innan beslut om sintidsbehandling?

- Nej, inga prov togs
- Ja, prov togs från vissa kor
- Ja, prov togs från alla kor

Denna informationsbox visas endast i läge förhandsgranskningen.

Följande kriterium måste vara uppfyllda för att följande fråga ska visas:

Om frågan Sintidsbehandlades vissa eller alla kor under senaste året? innehåller något av dessa svarsalternativ

- Vissa kor

47) Hur många av korna behandlades under senaste året?

- Enstaka kor
- Cirka en av fyra
- Cirka varannan
- Cirka tre av fyra

Denna informationsbox visas endast i läge förhandsgranskningen.

Följande kriterium måste vara uppfyllda för att följande fråga ska visas:

Om frågan Sintidsbehandlades vissa eller alla kor under senaste året? innehåller något av dessa svarsalternativ

- Vissa kor

48) VIlken/vilka av följande faktorer påverkade valet av vilka kor som skulle sintidsbehandlas? (flera alternativ möjliga)

☐ Om kon haft klinisk (synlig) mastit under laktationen
☐ Kons celltal vid sista provmjölkningen innan sinläggning (ange celltalsgräns i fältet nedan t ex över 200 000/ml)
☐ Kons juverhälsoklass (JHKL) vid sista provmjölkningen innan sinläggning (ange gräns för JHKL i fältet nedan t ex över JHKL3)
☐ CMT-reaktion i någon juverdel i samband med sinläggningen
☐ Bakteriefynd vid odling (ange vilka bakterier som leder till sintidsbehandling i fältet nedan)
☐ Annat, ange vad

Denna informationsbox visas endast i läge förhandsgranskningen.

Följande kriterium måste vara uppfyllda för att följande fråga ska visas:

Om frågan Sintidsbehandlades vissa eller alla kor under senaste året? innehåller något av dessa svarsalternativ

- Vissa kor

49) Vilka juvertuber (se bilder) användes under senaste året? (flera alternativ möjliga)

☐ Carepen
☐ Benestermycin
☐ Siccalactin
☐ Annat, ange vad

Denna informationsbox visas endast i läge förhandsgranskningen.

Följande kriterium måste vara uppfyllda för att följande fråga ska visas:

Om frågan Sintidsbehandlades vissa eller alla kor under senaste året? innehåller något av dessa svarsalternativ

- Vissa kor

50) Användes samma typ av juvertuber till de flesta behandlingarna (minst tre av fyra)?

○ Nej
○ Ja, ange vilken typ

Denna informationsbox visas endast i läge förhandsgranskningen.

Följande kriterium måste vara uppfyllda för att följande fråga ska visas:

Om frågan Användes samma typ av juvertuber till de flesta behandlingarna (minst tre av fyra)? innehåller något av dessa svarsalternativ

- Nej

51) Varför användes olika typer av juvertuber?

Denna informationsbox visas endast i läge förhandsgranskningen.

Följande kriterium måste vara uppfyllda för att följande fråga ska visas:

Om frågan Sintidsbehandlades vissa eller alla kor under senaste året? innehåller något av dessa svarsalternativ

- Vissa kor

52) Sintidsbehandlas alltid alla 4 juverdelarna?

- [ ] Nej
- [ ] Ja

Denna informationsbox visas endast i läge förhandsgranskningen.

Följande kriterium måste vara uppfyllda för att följande fråga ska visas:

Om frågan Sintidsbehandlas alltid alla 4 juverdelarna? innehåller något av dessa svarsalternativ

- Nej

53) Vilka juverdelar sintidsbehandlas?

- [ ] Endast de som mjölkas vid sinläggning
- [ ] Endast de som har högt CMT
- [ ] Annat, ange vad:

Denna informationsbox visas endast i läge förhandsgranskningen.

Följande kriterium måste vara uppfyllda för att följande fråga ska visas:

Om frågan Sintidsbehandlades vissa eller alla kor under senaste året? innehåller något av dessa svarsalternativ

- Vissa kor

54) Används alltid samma rutin när själva sintidsbehandlingen genomförs?

- [ ] Nej
- [ ] Ja

Denna informationsbox visas endast i läge förhandsgranskningen.

Följande kriterium måste vara uppfyllda för att följande fråga ska visas:

Om frågan Sintidsbehandlades vissa eller alla kor under senaste året? innehåller något av dessa svarsalternativ

- Vissa kor

55) Vilket/vilka av följande moment görs vanligen i samband med själva sintidsbehandlingen?

- [ ] Tvättar händerna innan behandling
- [ ] Använder rena handskar
- [ ] Torkar av spenarna med papper
- [ ] Torkar av spenarna med fuktad engångsduk
- [ ] Torkar rent spenspetsen med bifogad servett
- [ ] Torkar rent spenspetsen med bomull fuktad med alkohol
- [ ] Använder lång spets på juvertuben (dvs tar bort hela korken)
- [ ] Använder kort spets på juvertuben (dvs tar endast bort yttre änden av korken)
- [ ] Masserar spenen/juverdelen efter att innehållet i juvertuben sprutats in

☐ Annat, ange vad:

Denna informationsbox visas endast i läge förhandsgranskningen.

Följande kriterium måste vara uppfyllda för att följande fråga ska visas:

Om frågan Sintidsbehandlades vissa eller alla kor under senaste året? innehåller något av dessa svarsalternativ

- Vissa kor

56) Finns det några risker eller svårigheter med själva sintidsbehandlingen?

○ Nej

○ Ja, ange vilka:

Denna informationsbox visas endast i läge förhandsgranskningen.

Följande kriterium måste vara uppfyllda för att följande fråga ska visas:

Om frågan Sintidsbehandlades vissa eller alla kor under senaste året? innehåller något av dessa svarsalternativ

- Alla kor

57) Varför valde ni att behandla alla kor med sintidsbehandling? (flera alternativ möjliga)

☐ Besättningen har/har haft problem med Streptococcus agalactiae
☐ Rekommenderas av rådgivare
☐ Annat, ange vad

Denna informationsbox visas endast i läge förhandsgranskningen.

Följande kriterium måste vara uppfyllda för att följande fråga ska visas:

Om frågan Sintidsbehandlades vissa eller alla kor under senaste året? innehåller något av dessa svarsalternativ

- Alla kor

58) Tog ni mjölkprover för bakteriologisk undersökning innan beslut om sintidsbehandling?

○ Nej, inga prov togs

○ Ja, prov togs från vissa kor

○ Ja, prov togs från alla kor

Denna informationsbox visas endast i läge förhandsgranskningen.

Följande kriterium måste vara uppfyllda för att följande fråga ska visas:

Om frågan Sintidsbehandlades vissa eller alla kor under senaste året? innehåller något av dessa svarsalternativ

- Alla kor

59) Vilken/vilka juvertuber (se bilder) användes under senaste året? (flera alternativ möjliga)

☐ Carepen
☐ Benestermycin
☐ Siccalactin

☐ Annat, ange vad

Denna informationsbox visas endast i läge förhandsgranskningen.

Följande kriterium måste vara uppfyllda för att följande fråga ska visas:

Om frågan Sintidsbehandlades vissa eller alla kor under senaste året? innehåller något av dessa svarsalternativ

- Alla kor

60) Användes samma typ av juvertuber till de flesta behandlingarna (minst tre av fyra)?

- Nej
- Ja, ange vilken typ

Denna informationsbox visas endast i läge förhandsgranskningen.

Följande kriterium måste vara uppfyllda för att följande fråga ska visas:

Om frågan Användes samma typ av juvertuber till de flesta behandlingarna (minst tre av fyra)? innehåller något av dessa svarsalternativ

- Nej

61) Varför användes olika typer av juvertuber?

Denna informationsbox visas endast i läge förhandsgranskningen.

Följande kriterium måste vara uppfyllda för att följande fråga ska visas:

Om frågan Sintidsbehandlades vissa eller alla kor under senaste året? innehåller något av dessa svarsalternativ

- Alla kor

62) Sintidsbehandlas alltid alla 4 juverdelarna?

- Nej
- Ja

Denna informationsbox visas endast i läge förhandsgranskningen.

Följande kriterium måste vara uppfyllda för att följande fråga ska visas:

Om frågan Sintidsbehandlas alltid alla 4 juverdelarna? innehåller något av dessa svarsalternativ

- Nej

63) Vilka juverdelar behandlas?

☐ Endast de som mjölkas vid sinläggningen
☐ Endast de som har högt CMT
☐ Annat, ange vad:

Denna informationsbox visas endast i läge förhandsgranskningen.

Följande kriterium måste vara uppfyllda för att följande fråga ska visas:

Om frågan Sintidsbehandlades vissa eller alla kor under senaste året? innehåller något av dessa svarsalternativ

- Alla kor

64) Används alltid samma rutin när själva sintidsbehandlingen genomförs?

- Nej
- Ja

Denna informationsbox visas endast i läge förhandsgranskningen.

Följande kriterium måste vara uppfyllda för att följande fråga ska visas:

Om frågan Sintidsbehandlades vissa eller alla kor under senaste året? innehåller något av dessa svarsalternativ

- Alla kor

65) Vilket/vilka av följande moment görs vanligen i samband med själva sintidsbehandlingen?

- [ ] Tvättar händerna innan behandling
- [ ] Använder rena handskar
- [ ] Torkar av spenarna med papper
- [ ] Torkar av spenarna med fuktad engångsduk
- [ ] Torkar av spenspetsen med bifogad servett
- [ ] Torkar av spenspetsen med bomull fuktad med alkohol
- [ ] Använder lång spets på juvertuben (dvs tar bort hela korken)
- [ ] Använder kort spets på juvertuben (dvs tar endast bort yttre änden av korken)
- [ ] Annat, ange vad

Denna informationsbox visas endast i läge förhandsgranskningen.

Följande kriterium måste vara uppfyllda för att följande fråga ska visas:

Om frågan Sintidsbehandlades vissa eller alla kor under senaste året? innehåller något av dessa svarsalternativ

- Alla kor

66) Finns det några risker eller svårigheter med själva sintidsbehandlingen?

- Nej
- Ja, ange vilka:

Denna informationsbox visas endast i läge förhandsgranskningen.

Följande kriterium måste vara uppfyllda för att följande fråga ska visas:

Om frågan Används sintidsbehandling med antibiotika i besättningen? innehåller något av dessa svarsalternativ

- Ja

67) Hur ofta brukar du bedöma sintidsbehandlingens effekt genom att (ange ett alternativ per rad)

	Aldrig/nästan aldrig	Mindre ofta	Ganska ofta	Alltid/nästan alltid
Kontrollera celltalet vid första provmjölkningen efter kalvning	☐	☐	☐	☐
Göra CMT-undersökning efter kalvning	☐	☐	☐	☐
Ta mjölkprov för bakteriologisk undersökning efter kalvning	☐	☐	☐	☐
Annat, ange vad i raden nedan	☐	☐	☐	☐

Denna informationsbox visas endast i läge förhandsgranskningen.

Följande kriterium måste vara uppfyllda för att följande fråga ska visas:

Om frågan Används sintidsbehandling med antibiotika i besättningen? innehåller något av dessa svarsalternativ

- Ja

69) Hur anser du att sintidsbehandling med antibiotika påverkar följande aspekter på djurens hälsa och produktion under början av kommande laktation? (ange ett alternativ per rad)

	Försämras	Ingen effekt	Förbättras	Vet ej
Kons juverhälsa	☐	☐	☐	☐
Kalvens hälsa	☐	☐	☐	☐
Kons mjölkproduktion	☐	☐	☐	☐
Kons livslängd	☐	☐	☐	☐

FRÅGOR OM BEHANDLING MED SPENFÖRSLUTARE

Definition: Behandling med spenförslutare innebär att juvertuber med vismutsubnitrat (inte antibiotika) sprutas in i alla juverdelar via spenkanalen efter sista mjölkningen under sinläggningen dvs precis innan sinperioden tar vid.

71) Används spenförslutare i besättningen?

☐ Nej

☐ Ja

Denna informationsbox visas endast i läge förhandsgranskningen.

Följande kriterium måste vara uppfyllda för att följande fråga ska visas:

Om frågan Används spenförslutare i besättningen? innehåller något av dessa svarsalternativ

- Nej

72) Varför används inte spenförslutare i besättningen? (flera alternativ möjliga)

- [ ] Korna har så god juverhälsa att det inte behövs
- [ ] Behandlingen är för dyr
- [ ] Behandlingen är för arbetskrävande
- [ ] Annat, ange varför

Denna informationsbox visas endast i läge förhandsgranskningen.

Följande kriterium måste vara uppfyllda för att följande fråga ska visas:

Om frågan Används spenförslutare i besättningen? innehåller något av dessa svarsalternativ

- Ja

73) Varför används spenförslutare? (flera alternativ möjliga)

- [ ] På grund av juverhälsoproblem i besättningen
- [ ] Rekommenderas av rådgivare
- [ ] Annat, ange vad

Denna informationsbox visas endast i läge förhandsgranskningen.

Följande kriterium måste vara uppfyllda för att följande fråga ska visas:

Om frågan Används spenförslutare i besättningen? innehåller något av dessa svarsalternativ

- Ja

74) Behandlades vissa eller alla kor under senaste året?

- ( ) Vissa
- ( ) Alla

Denna informationsbox visas endast i läge förhandsgranskningen.

Följande kriterium måste vara uppfyllda för att följande fråga ska visas:

Om frågan Behandlades vissa eller alla kor under senaste året? innehåller något av dessa svarsalternativ

- Vissa

75) Tog ni mjölkprover för bakteriologisk undersökning innan beslut om behandling?

- ( ) Nej, inga prov togs
- ( ) Ja, prov togs från vissa kor
- ( ) Ja, prov togs från alla kor

Denna informationsbox visas endast i läge förhandsgranskningen.

Följande kriterium måste vara uppfyllda för att följande fråga ska visas:

Om frågan Behandlades vissa eller alla kor under senaste året? innehåller något av dessa svarsalternativ

- Vissa

76) Hur många av korna behandlades under senaste året?

- Enstaka kor
- Cirka en av fyra kor
- Cirka varannan ko
- Cirka tre av fyra kor

Denna informationsbox visas endast i läge förhandsgranskningen.

Följande kriterium måste vara uppfyllda för att följande fråga ska visas:

Om frågan Behandlades vissa eller alla kor under senaste året? innehåller något av dessa svarsalternativ

- Vissa

77) Finns det några risker eller svårigheter med själva behandlingen?

- Nej
- Ja, ange vilka:

Denna informationsbox visas endast i läge förhandsgranskningen.

Följande kriterium måste vara uppfyllda för att följande fråga ska visas:

Om frågan Behandlades vissa eller alla kor under senaste året? innehåller något av dessa svarsalternativ

- Alla

78) Tog ni mjölkprover för bakteriologisk undersökning innan beslut om behandling?

- Nej, inga prov togs
- Ja, prov togs från vissa kor
- Ja, prov togs från alla kor

Denna informationsbox visas endast i läge förhandsgranskningen.

Följande kriterium måste vara uppfyllda för att följande fråga ska visas:

Om frågan Behandlades vissa eller alla kor under senaste året? innehåller något av dessa svarsalternativ

- Alla

79) Varför valde ni att behandla alla kor? (flera alternativ möjliga)

- [ ] Besättningen har/har haft problem med E coli och/eller Klebsiella
- [ ] Rekommenderas av rådgivare
- [ ] Annat, ange vad

Denna informationsbox visas endast i läge förhandsgranskningen.

Följande kriterium måste vara uppfyllda för att följande fråga ska visas:

Om frågan Behandlades vissa eller alla kor under senaste året? innehåller något av dessa svarsalternativ

- Alla

80) Finns det några risker eller svårigheter med själva behandlingen?

- Nej
- Ja, ange vilka:

Denna informationsbox visas endast i läge förhandsgranskningen.

Följande kriterium måste vara uppfyllda för att följande fråga ska visas:

Om frågan Används spenförslutare i besättningen? innehåller något av dessa svarsalternativ

- Ja

81) Används spenförslutare i kombination med sintidsantibiotika?

- Nej
- Ja, ibland
- Ja, alltid

Denna informationsbox visas endast i läge förhandsgranskningen.

Följande kriterium måste vara uppfyllda för att följande fråga ska visas:

Om frågan Används spenförslutare i kombination med sintidsantibiotika? innehåller något av dessa svarsalternativ

- Ja, alltid
- Ja, ibland

82) Varför kombineras spenförslutare och sintidsantibiotika?

- På grund av juverhälsoproblem i besättningen?
- Rekommenderas av rådgivare
- Annat, ange vad:

Denna informationsbox visas endast i läge förhandsgranskningen.

Följande kriterium måste vara uppfyllda för att följande fråga ska visas:

Om frågan Används spenförslutare i besättningen? innehåller något av dessa svarsalternativ

- Ja

83) Hur ofta brukar du bedöma behandlingens effekt genom att (ange ett alternativ per rad)

	Aldrig/nästan aldrig	Mindre ofta	Ganska ofta	Alltid/nästan alltid
Kontrollera celltalet vid första provmjölkningen efter kalvning	☐	☐	☐	☐
Göra CMT-undersökning efter kalvning	☐	☐	☐	☐
Ta mjölkprov för bakteriologisk undersökning efter kalvning	☐	☐	☐	☐
Annat, ange vad på raden nedan	☐	☐	☐	☐

Denna informationsbox visas endast i läge förhandsgranskningen.

Följande kriterium måste vara uppfyllda för att följande fråga ska visas:

Om frågan Används spenförslutare i besättningen? innehåller något av dessa svarsalternativ

- Ja

Denna informationsbox visas endast i läge förhandsgranskningen.

Följande kriterium måste vara uppfyllda för att följande fråga ska visas:

Om frågan Används spenförslutare i besättningen? innehåller något av dessa svarsalternativ

- Ja

85) Hur anser du att behandling med spenförslutare påverkar följande aspekter på djurens hälsa och produktion under början av kommande laktation? (ange ett alternativ per rad)

	Försämras	Ingen effekt	Förbättras	Vet ej
Kons juverhälsa	☐	☐	☐	☐
Kalvens hälsa	☐	☐	☐	☐
Kons mjölkproduktion	☐	☐	☐	☐
Kons livslängd	☐	☐	☐	☐

FRÅGOR OM SINPERIODEN

Definition: Sinperioden är tiden mellan sista mjölkningen i en laktation och kalvning.

87) Har ni skrivna rutiner för sinperioden?

☐ Nej

☐ Ja

88) Hur många personer tar hand om korna under sinperioden?

☐ 1

☐ 2-3

☐ Fler än 3

89) Är sinperioden lika lång (t ex 8 veckor) för de flesta (minst tre av fyra) korna?

☐ Nej

☐ Ja

Denna informationsbox visas endast i läge förhandsgranskningen.

Följande kriterium måste vara uppfyllda för att följande fråga ska visas:

Om frågan Är sinperioden lika lång (t ex 8 veckor) för de flesta (minst tre av fyra) korna? innehåller något av dessa svarsalternativ

- Nej

90) Hur många av alla kor har en sinperiod som är (ange ett svar per rad)

	Inga	Färre än hälften	Cirka hälften	Fler än hälften
Kortare än 6 veckor	☐	☐	☐	☐
6-8 veckor	☐	☐	☐	☐
Längre än 8 veckor	☐	☐	☐	☐

Denna informationsbox visas endast i läge förhandsgranskningen.

Följande kriterium måste vara uppfyllda för att följande fråga ska visas:

Om frågan Är sinperioden lika lång (t ex 8 veckor) för de flesta (minst tre av fyra) korna? innehåller något av dessa svarsalternativ

- Nej

91) Varför varierar sinperiodens längd mellan korna?

Denna informationsbox visas endast i läge förhandsgranskningen.

Följande kriterium måste vara uppfyllda för att följande fråga ska visas:

Om frågan Är sinperioden lika lång (t ex 8 veckor) för de flesta (minst tre av fyra) korna? innehåller något av dessa svarsalternativ

- Ja

92) Hur lång är sinperioden?

☐ Kortare än 6 veckor

☐ 6-8 veckor

☐ Längre än 8 veckor

☐ Annan tid, ange vad

93) Går sinkorna med mjölkande kor hela eller delar av sinperioden?

☐ Nej

☐ Ja, ganska ofta/Ibland (färre än tre av fyra kor)

☐ Ja, för det mesta (minst tre av fyra kor)

Denna informationsbox visas endast i läge förhandsgranskningen.

Följande kriterium måste vara uppfyllda för att följande fråga ska visas:

Om frågan Går sinkorna med mjölkande kor hela eller delar av sinperioden? innehåller något av dessa svarsalternativ

- Nej

94) Var finns sinkorna?

☐ I egen avdelning i samma stall som mjölkkorna
☐ I annat stall
☐ Annat, ange var

95) Flyttas sinkorna mellan olika avdelningar under sinperioden?

- Nej
- Ja, ganska ofta/ibland (färre än tre av fyra kor)
- Ja, oftast (minst tre av fyra kor)

Denna informationsbox visas endast i läge förhandsgranskningen.

Följande kriterium måste vara uppfyllda för att följande fråga ska visas:

Om frågan Flyttas sinkorna mellan olika avdelningar under sinperioden? innehåller något av dessa svarsalternativ

- Ja, ganska ofta/ibland (färre än tre av fyra kor)

96) Hur många av korna flyttas olika många gånger under sinperioden? (ange ett alternativ per rad)

	Inga	Färre än hälften	Cirka hälften	Fler än hälften
1-2 gånger				
Fler än 2 gånger				

Denna informationsbox visas endast i läge förhandsgranskningen.

Följande kriterium måste vara uppfyllda för att följande fråga ska visas:

Om frågan Flyttas sinkorna mellan olika avdelningar under sinperioden? innehåller något av dessa svarsalternativ

- Ja, oftast (minst tre av fyra kor)

97) Hur många gånger flyttas korna under sinperioden?

- 1-2 gånger
- Fler än 2 gånger

98) Spendoppas/-sprejas de flesta korna (minst tre av fyra) under sinperioden?

- Nej
- Ja, ange när (t ex första o sista veckan)

99) Undersöks juvret (t ex tittar, känner, CMT-paddlar) på de flesta korna (minst tre av fyra) under sinperioden?

- Nej
- Ja

Denna informationsbox visas endast i läge förhandsgranskningen.

Följande kriterium måste vara uppfyllda för att följande fråga ska visas:

Om frågan Undersöks juvret (t ex tittar, känner, CMT-paddlar) på de flesta korna (minst tre av fyra) under sinperioden? innehåller något av dessa svarsalternativ

- Ja

100) Hur undersöks juvret under sinperioden?

- Tittar på juvret
- Känner på juvret
- CMT-paddlar
- Annat, ange vad

Denna informationsbox visas endast i läge förhandsgranskningen.

Följande kriterium måste vara uppfyllda för att följande fråga ska visas:

Om frågan Undersöks juvret (t ex tittar, känner, CMT-paddlar) på de flesta korna (minst tre av fyra) under sinperioden? innehåller något av dessa svarsalternativ

- Ja

101) När undersöks juvret under sinperioden (t ex första/sista veckan)?

102) Använder ni några andra preparat (utom spendopp/-sprej, sintidsantibiotika eller spenförslutare) under sinperioden?

- Nej
- Ja, homeopatiska preparat
- Ja, annat, ange vad och hur

Denna informationsbox visas endast i läge förhandsgranskningen.

Följande kriterium måste vara uppfyllda för att följande fråga ska visas:

Om frågan Använder ni några andra preparat (utom spendopp/-sprej, sintidsantibiotika eller spenförslutare) under sinperioden? innehåller något av dessa svarsalternativ

- Ja, homeopatiska preparat

103) Till hur många av sinkorna används homeopatiska preparat?

- Färre än hälften
- Cirka hälften
- Fler än hälften

104) Anser du att sinperioden är viktig för följande aspekter på djurens hälsa och produktion under början av kommande laktation? (ange ett alternativ per rad)

	Nej	Ja	Vet ej
Kons juverhälsa	☐	☐	☐
Kalvens hälsa	☐	☐	☐
Kons mjölkproduktion	☐	☐	☐
Kons fruktsamhet	☐	☐	☐
Förekomst av kalvningsförlamning	☐	☐	☐
Förekomst av foderleda hos korna	☐	☐	☐

FRÅGOR OM INFORMATION OCH RÅDGIVNING

122) Har du fått rådgivning rörande sinläggning och sintidsbehandling någon gång?

☐ Nej

☐ Ja, under senaste året

☐ Ja, under de senaste fem åren

☐ Ja, tidigare

Denna informationsbox visas endast i läge förhandsgranskningen.

Följande kriterium måste vara uppfyllda för att följande fråga ska visas:

Om frågan Har du fått rådgivning rörande sinläggning och sintidsbehandling någon gång? innehåller något av dessa svarsalternativ

- Ja, tidigare
- Ja, under de senaste fem åren
- Ja, under senaste året

123) Av vilken/vilka organisation/er eller liknande fick du sådan rådgivning?

124) Skulle du vilja få information/rådgivning om sinläggning och sintidsbehandling i framtiden?

☐ Nej

☐ Ja, men endast om det inte kostar någor

☐ Ja, oavsett om det kostar något eller inte

Supplementary Document S2. Questionnaire (in Swedish) presented to Swedish cattle veterinarians on advice given on dry cow therapy with antibiotics and treatment with internal teat sealants

Sinläggning, sintidsbehandling och sinperiod - enkät till veterinärer

Syftet med denna enkät är att undersöka vilka råd svenska veterinärer ger till mjölkproducenter och deras personal avseende rutiner för sinläggning och sintidsbehandling av mjölkkor samt för sinperioden. En liknande enkät skickas även till mjölkproducenter i landet. Resultaten kommer att bli tillgängliga för alla och svaren ska ligga till grund för bedömning av behov av information om dessa områden. Vi är därför mycket tacksamma för att du vill fylla i enkäten som tar cirka 10-15 minuter. Enkäten är anonym.

Projektet är ett samarbete mellan Statens veterinärmedicinska anstalt (SVA) och Växa Sverige.

Om du har några frågor kan du på dagtid nå projektledare Karin Persson Waller, SVA, via epost (karin.persson-waller@sva.se) eller telefon (018-674672).

Din identitet kommer att vara dold.

När dold identitet är aktiverat i enkäten, sparas inte identifierbar information såsom: webbläsarens version, IP-adress, operativsystem eller e-postadress i svaren. Detta för att skydda respondenternas identitet.

GRUNDLÄGGANDE FRÅGOR

2) Vilket år tog du veterinärexamen? (ange t ex 1980, 2015)

3) I vilket land tog du veterinärexamen?

- ☐ Sverige
- ☐ Annat, ange vilket

4) I vilket/vilka län arbetar du huvudsakligen?

- ☐ Stockholms län
- ☐ Uppsala län
- ☐ Södermanlands län
- ☐ Östergötlands län
- ☐ Jönköpings län
- ☐ Kronobergs län
- ☐ Kalmar län
- ☐ Gotlands län
- ☐ Blekinge län
- ☐ Skåne län
- ☐ Hallands län
- ☐ Västra Götalands län
- ☐ Värmlands län
- ☐ Örebro län
- ☐ Västmanlands län
- ☐ Dalarnas län
- ☐ Gävleborgs län
- ☐ Västernorrlands län
- ☐ Jämtlands län

☐ Västerbottens län
☐ Norrbottens län

5) Är du kvinna eller man?

- ○ Kvinna
- ○ Man
- ○ Vill inte svara

6) Har du vidareutbildat dig inom nötkreaturens sjukdomar?

- ○ Nej
- ◉ Ja

Denna informationsbox visas endast i läge förhandsgranskningen.

Följande kriterium måste vara uppfyllda för att följande fråga ska visas:

Om frågan Har du vidareutbildat dig inom nötkreaturens sjukdomar? innehåller något av dessa svarsalternativ

- Ja

7) Hur har du vidareutbildat dig inom nötkreaturens sjukdomar?

☐ Genom Hälsopaket mjölk
☐ På annat sätt, ange vad
rundläggande frågor

8) Hur många år har du arbetat med nötkreaturspraktik?

9) Ungefär hur många mastiter behandlar du per månad?

- ○ Färre än 1
- ○ 1-3
- ○ 4-8
- ○ 9-15
- ○ Fler än 15

FRÅGOR OM SINLÄGGNINGEN

Definition: Sinläggningen är den period i slutet på laktationen då man förbereder kon innan hon går i sin dvs innan man helt slutar att mjölka kon.

11) Brukar du ge råd om sinläggningsrutiner till mjölkproducenter och/eller deras personal?

- ○ Nej, sällan/aldrig
- ○ Ja, ibland

Ja, ofta

Denna informationsbox visas endast i läge förhandsgranskningen.

Följande kriterium måste vara uppfyllda för att följande fråga ska visas:

Om frågan Brukar du ge råd om sinläggningsrutiner till mjölkproducenter och/eller deras personal? innehåller något av dessa svarsalternativ

- Nej, sällan/aldrig

12) Varför brukar du inte ge råd om sinläggningsrutiner? (flera alternativ möjliga)

- Anser mig ha för liten kunskap
- Tidsbrist
- Upplever inte att det efterfrågas
- Annat, ange vad

13) Anser du att något/några av följande områden är viktiga vid sinläggningen? (ett svar per rad)

	Nej	Ja, ganska viktigt	Ja, mycket viktigt	Vet ej
Mjölkningen				
Utfodringen				
Vattentillförseln				
Hygienen i kons närmiljö				
Förflyttning av korna				
Spendoppning/spensprejning				
Undersökning av juvret				

Denna informationsbox visas endast i läge förhandsgranskningen.

Följande kriterium måste vara uppfyllda för att följande fråga ska visas:

Om frågan Mjölkningen innehåller något av dessa svarsalternativ

- Ja, mycket viktigt
- Ja, ganska viktigt

14) Rekommenderar du att mjölkningen avslutas tvärt?

Nej

Ja, under vissa förutsättningar

Ja, alltid

Denna informationsbox visas endast i läge förhandsgranskningen.

Följande kriterium måste vara uppfyllda för att följande fråga ska visas:

Om frågan Rekommenderar du att mjölkningen avslutas tvärt? innehåller något av dessa svarsalternativ

- Ja, under vissa förutsättningar

15) * Under vilka förutsättningar rekommenderar du att mjölkningen avslutas tvärt?

Denna informationsbox visas endast i läge förhandsgranskningen.

Följande kriterium måste vara uppfyllda för att följande fråga ska visas:

Om frågan Mjölkningen innehåller något av dessa svarsalternativ

- Ja, mycket viktigt
- Ja, ganska viktigt

16) Rekommenderar du mjölkning med förlängt intervall under sinläggningen?

- [x] Nej
- [ ] Ja, under vissa förutsättningar
- [ ] Ja, alltid

Denna informationsbox visas endast i läge förhandsgranskningen.

Följande kriterium måste vara uppfyllda för att följande fråga ska visas:

Om frågan Rekommenderar du mjölkning med förlängt intervall under sinläggningen? innehåller något av dessa svarsalternativ

- Ja, under vissa förutsättningar

17) * Under vilka förutsättningar rekommenderar du mjölkning med förlängt intervall?

Denna informationsbox visas endast i läge förhandsgranskningen.

Följande kriterium måste vara uppfyllda för att följande fråga ska visas:

Om frågan Rekommenderar du mjölkning med förlängt intervall under sinläggningen? innehåller något av dessa svarsalternativ

- Ja, alltid
- Ja, under vissa förutsättningar

18) Hur ofta rekommenderar du att korna mjölkas under sinläggningen?

- [ ] En gång per dag
- [ ] En gång varannan dag
- [ ] Annat intervall, ange vad

Denna informationsbox visas endast i läge förhandsgranskningen.

Följande kriterium måste vara uppfyllda för att följande fråga ska visas:

Om frågan Rekommenderar du mjölkning med förlängt intervall under sinläggningen? innehåller något av dessa svarsalternativ

- Ja, alltid
- Ja, under vissa förutsättningar

19) Hur länge rekommenderar du att korna mjölkas med förlängt intervall under sinläggningen?

- ☐ Cirka 1 vecka
- ☐ Cirka 2 veckor
- ☐ Annat, ange antal veckor

Denna informationsbox visas endast i läge förhandsgranskningen.

Följande kriterium måste vara uppfyllda för att följande fråga ska visas:

Om frågan Utfodringen innehåller något av dessa svarsalternativ

- Ja, mycket viktigt
- Ja, ganska viktigt

20) Vilka råd om utfodringen brukar du ge? (ett svar per rad)

	Minskas	Är oförändrad	Ökas
Att totala mängden kraftfoder	☐	☐	☐
Att totala mängden grovfoder	☐	☐	☐
Att mängden halm	☐	☐	☐
Att mängden hö	☐	☐	☐
Att mängden ensilage	☐	☐	☐

Denna informationsbox visas endast i läge förhandsgranskningen.

Följande kriterium måste vara uppfyllda för att följande fråga ska visas:

Om frågan Utfodringen innehåller något av dessa svarsalternativ

- Ja, mycket viktigt
- Ja, ganska viktigt

21) Ange eventuella andra råd om utfodring (t ex byte av foderparti mm)

Denna informationsbox visas endast i läge förhandsgranskningen.

Följande kriterium måste vara uppfyllda för att följande fråga ska visas:

Om frågan Utfodringen innehåller något av dessa svarsalternativ

- Ja, mycket viktigt
- Ja, ganska viktigt

22) Hur länge rekommenderar du att korna får sinläggningsfoderstat?

- ☐ Cirka 1 vecka
- ☐ Cirka 2 veckor
- ☒ Annat, ange antal veckor

Denna informationsbox visas endast i läge förhandsgranskningen.

Följande kriterium måste vara uppfyllda för att följande fråga ska visas:

Om frågan Vattentillförseln innehåller något av dessa svarsalternativ

- Ja, mycket viktigt
- Ja, ganska viktigt

23) Vilka råd om vattentillförseln brukar du ge?

- Att vattnet tas bort helt
- Att mängden minskas
- Att mängden är oförändrad
- Att mängden ökas
- Annat, ange vad

Denna informationsbox visas endast i läge förhandsgranskningen.

Följande kriterium måste vara uppfyllda för att följande fråga ska visas:

Om frågan Hygienen i kons närmiljö innehåller något av dessa svarsalternativ

- Ja, mycket viktigt
- Ja, ganska viktigt

24) * Vilka råd om hygienen i kons närmiljö brukar du ge?

Denna informationsbox visas endast i läge förhandsgranskningen.

Följande kriterium måste vara uppfyllda för att följande fråga ska visas:

Om frågan Förflyttning av korna innehåller något av dessa svarsalternativ

- Ja, mycket viktigt
- Ja, ganska viktigt

25) Rekommenderar du att korna flyttas från gruppen med de mjölkande korna under sinläggningen?

- Nej
- Ja
- Annat, ange vad

Denna informationsbox visas endast i läge förhandsgranskningen.

Följande kriterium måste vara uppfyllda för att följande fråga ska visas:

Om frågan Spendoppning/spensprejning innehåller något av dessa svarsalternativ

- Ja, mycket viktigt
- Ja, ganska viktigt

26) Hur ofta rekommenderar du att korna spendoppas/spensprejas under sinläggningen?

- Efter varje mjölkning
- Vid fasta tider under dygnet
- Annat, ange hur ofta

Denna informationsbox visas endast i läge förhandsgranskningen.

Följande kriterium måste vara uppfyllda för att följande fråga ska visas:

Om frågan Undersökning av juvret innehåller något av dessa svarsalternativ

- Ja, mycket viktigt
- Ja, ganska viktigt

27) Vilka juverundersökningar rekommenderar du under sinläggningen? (flera alternativ möjliga)

- Att titta på juvret
- Att känna på juvret
- Att göra CMT-undersökning
- Annat, ange vad

28) Anser du att sinläggningsrutinerna är viktiga för följande aspekter på kornas hälsa och produktion under början av kommande laktation? (ange ett alternativ per rad)

	Nej	Ja, ganska viktiga	Ja, mycket viktiga	Vet ej
Kons juverhälsa				
Kons mjölkproduktion				
Kons fruktsamhet				

FRÅGOR OM SINTIDSBEHANDLING MED ANTIBIOTIKA

Definition: Sintidsbehandling med antibiotika innebär att antibiotika (oftast långtidsverkande) sprutas in i alla juverdelar via spenkanalen efter sista mjölkningen under sinläggningen dvs precis innan sinperioden tar vid.

30) Hur ofta brukar du skriva ut juvertuber för sintidsbehandling?

- Varje vecka
- Någon gång i månaden
- Mer sällan
- Aldrig

Denna informationsbox visas endast i läge förhandsgranskningen.

Följande kriterium måste vara uppfyllda för att följande fråga ska visas:

Om frågan Hur ofta brukar du skriva ut juvertuber för sintidsbehandling? innehåller något av dessa svarsalternativ

- Mer sällan

- Någon gång i månaden
- Varje vecka

31) Brukar du använda dig av bakteriologisk diagnostik innan du ordinerar sintidsbehandling?

- Sällan/aldrig
- Mindre ofta
- Ganska ofta
- Alltid/nästan alltid

Denna informationsbox visas endast i läge förhandsgranskningen.

Följande kriterium måste vara uppfyllda för att följande fråga ska visas:

Om frågan Brukar du använda dig av bakteriologisk diagnostik innan du ordinerar sintidsbehandling? innehåller något av dessa svarsalternativ

- Alltid/nästan alltid
- Ganska ofta
- Mindre ofta

32) Var sker den bakteriologiska diagnostiken?

- Egen odling
- På ackrediterat labb
- Annat, ange vad

Denna informationsbox visas endast i läge förhandsgranskningen.

Följande kriterium måste vara uppfyllda för att följande fråga ska visas:

Om frågan Hur ofta brukar du skriva ut juvertuber för sintidsbehandling? innehåller något av dessa svarsalternativ

- Mer sällan
- Någon gång i månaden
- Varje vecka

33) Brukar du oftast skriva ut samma sorts juvertuber vid sintidsbehandling?

- Nej
- Ja

Denna informationsbox visas endast i läge förhandsgranskningen.

Följande kriterium måste vara uppfyllda för att följande fråga ska visas:

Om frågan Brukar du oftast skriva ut samma sorts juvertuber vid sintidsbehandling? innehåller något av dessa svarsalternativ

- Ja

34) Vilket preparat brukar du skriva ut?

- Benestermycin
- Carepen

- [ ] Siccalactin
- [ ] Annat, ange vad

Denna informationsbox visas endast i läge förhandsgranskningen.

Följande kriterium måste vara uppfyllda för att följande fråga ska visas:

Om frågan Brukar du oftast skriva ut samma sorts juvertuber vid sintidsbehandling? innehåller något av dessa svarsalternativ

- Nej

35) Till hur stor andel av korna skriver du ut följande juvertuber vid sintidsbehandling? (ett svar per rad)

	Inga	Färre än hälften	Fler än hälften
Benestermycin	☐	☐	☐
Carepen	☐	☐	☐
Siccalactin	☐	☐	☐
Annat, ange vad i rutan nedan	☐	☐	☐

Denna informationsbox visas endast i läge förhandsgranskningen.

Följande kriterium måste vara uppfyllda för att följande fråga ska visas:

Om frågan Brukar du oftast skriva ut samma sorts juvertuber vid sintidsbehandling? innehåller något av dessa svarsalternativ

- Nej

Denna informationsbox visas endast i läge förhandsgranskningen.

Följande kriterium måste vara uppfyllda för att följande fråga ska visas:

Om frågan Hur ofta brukar du skriva ut juvertuber för sintidsbehandling? innehåller något av dessa svarsalternativ

- Mer sällan
- Någon gång i månaden
- Varje vecka

37) Hur ofta rekommenderar du sintidsbehandling till ALLA eller VISSA kor i en besättning? (ett svar per rad)

	Aldrig/nästan aldrig	Mindre ofta	Ganska ofta	Alltid/nästan alltid
Alla kor	☐	☐	☐	☐
Vissa kor	☐	☐	☐	☐

Denna informationsbox visas endast i läge förhandsgranskningen.

Följande kriterium måste vara uppfyllda för att följande fråga ska visas:

Om frågan Alla kor innehåller något av dessa svarsalternativ

- Alltid/nästan alltid
- Ganska ofta
- Mindre ofta

38) Varför väljer du ibland/alltid att behandla alla kor i en besättning?

☐ Besättningen har/har haft problem med Streptococcus agalactiae
☐ Annat, ange vad

Denna informationsbox visas endast i läge förhandsgranskningen.

Följande kriterium måste vara uppfyllda för att följande fråga ska visas:

Om frågan Vissa kor innehåller något av dessa svarsalternativ

- Alltid/nästan alltid
- Ganska ofta
- Mindre ofta

39) Vilken/vilka av följande faktorer påverkar ditt val av kor om du vill sintidsbehandla vissa kor i besättningen? (flera alternativ möjliga)

☐ Om kon haft klinisk (synlig) mastit under laktationen
☐ Kons celltal vid sista provmjölkningen innan sinläggning (ange celltalsgräns i fältet nedan t ex över 200 000/ml)
☐ Kons juverhälsoklass (JHKL) vid sista provmjölkningen innan sinläggning (ange gräns för JHKL i fältet nedan t ex över JHKL3)
☐ CMT-reaktion i någon juverdel i samband med sinläggningen
☐ Bakteriefynd vid odling (ange vilka bakterier som leder till sintidsbehandling i fältet nedan)
☐ Annat, ange vad

Denna informationsbox visas endast i läge förhandsgranskningen.

Följande kriterium måste vara uppfyllda för att följande fråga ska visas:

Om frågan Vissa kor innehåller något av dessa svarsalternativ

- Alltid/nästan alltid
- Ganska ofta
- Mindre ofta

Denna informationsbox visas endast i läge förhandsgranskningen.

Följande kriterium måste vara uppfyllda för att följande fråga ska visas:

Om frågan Hur ofta brukar du skriva ut juvertuber för sintidsbehandling? innehåller något av dessa svarsalternativ

- Mer sällan
- Någon gång i månaden
- Varje vecka

41) Rekommenderar du alltid att alla fyra juverdelarna behandlas?

☐ Nej

☐ Ja

Denna informationsbox visas endast i läge förhandsgranskningen.

Följande kriterium måste vara uppfyllda för att följande fråga ska visas:

Om frågan Rekommenderar du alltid att alla fyra juverdelarna behandlas? innehåller något av dessa svarsalternativ

- Nej

42) Vilka juverdelar rekommenderar du ska sintidsbehandlas?

☐ Endast de som mjölkas vid sinläggningen
☐ Endast de som har högt CMT
☐ Annat, ange vilka

Denna informationsbox visas endast i läge förhandsgranskningen.

Följande kriterium måste vara uppfyllda för att följande fråga ska visas:

Om frågan Hur ofta brukar du skriva ut juvertuber för sintidsbehandling? innehåller något av dessa svarsalternativ

- Mer sällan
- Någon gång i månaden
- Varje vecka

43) Brukar du ge råd om hur själva sintidsbehandlingen bör genomföras?

☐ Nej

☐ Ja, ibland

☐ Ja, ofta

Denna informationsbox visas endast i läge förhandsgranskningen.

Följande kriterium måste vara uppfyllda för att följande fråga ska visas:

Om frågan Brukar du ge råd om hur själva sintidsbehandlingen bör genomföras? innehåller något av dessa svarsalternativ

- Nej

44) Varför brukar du inte ge råd om hur själva sintidsbehandlingen bör genomföras? (flera val möjliga)

☐ Anser mig ha för liten kunskap
☐ Tidsbrist
☐ Upplever inte att det efterfrågas
☐ Annat, ange vad

Denna informationsbox visas endast i läge förhandsgranskningen.

Följande kriterium måste vara uppfyllda för att följande fråga ska visas:

Om frågan Brukar du ge råd om hur själva sintidsbehandlingen bör genomföras? innehåller något av dessa svarsalternativ

- Ja, ibland

45) * Vid vilka tillfällen brukar du ge råd om hur själva sintidsbehandlingen bör genomföras?

Denna informationsbox visas endast i läge förhandsgranskningen.

Följande kriterium måste vara uppfyllda för att följande fråga ska visas:

Om frågan Brukar du ge råd om hur själva sintidsbehandlingen bör genomföras? innehåller något av dessa svarsalternativ

- Ja, ofta
- Ja, ibland

46) Vilket/vilka av följande moment tycker du bör ingå i en god rutin vid sintidsbehandlingen? (flera alternativ möjliga)

☐ Tvätta händerna innan behandling
☐ Använda rena handskar
☐ Torka av spenarna med papper
☐ Torka av spenarna med fuktad engångsduk
☐ Torka rent spenspetsen med bifogad servett
☐ Torka rent spenspetsen med bomull fuktad med alkohol
☐ Använda lång spets (dvs ta bort hela korken) på juvertuben
☐ Använda kort spets (dvs endast ta bort yttre änden av korken) på juvertuben
☐ Massera spenen/juverdelen efter att innehållet i juvertuben sprutats in i spenen
☐ Annat, ange vad

Denna informationsbox visas endast i läge förhandsgranskningen.

Följande kriterium måste vara uppfyllda för att följande fråga ska visas:

Om frågan Hur ofta brukar du skriva ut juvertuber för sintidsbehandling? innehåller något av dessa svarsalternativ

- Mer sällan
- Någon gång i månaden
- Varje vecka

47) Finns det några risker eller svårigheter med sintidsbehandling?

◉ Nej

○ Ja, ange vilka

48) Känner du till att det finns lagstiftning om sintidsbehandling?

○ Nej

○ Ja

Denna informationsbox visas endast i läge förhandsgranskningen.

Följande kriterium måste vara uppfyllda för att följande fråga ska visas:

Om frågan Hur ofta brukar du skriva ut juvertuber för sintidsbehandling? innehåller något av dessa svarsalternativ

- Mer sällan
- Någon gång i månaden
- Varje vecka

49) Enligt SJVFS 2019:32 (D9) får veterinär, utan föregående undersökning av djur eller djurgrupp, förskriva antibiotikaläkemedel för lokal användning i juver inför sinläggning av kor endast om veterinären har kännedom om det enskilda djuret och det bakteriologiska hälsoläget i besättningen och besättningen deltar i ett strategiskt juverhälsoarbete. Hur ofta följer du denna lagstiftning?

- Sällan/aldrig
- Mindre ofta
- Ganska ofta
- Alltid/nästan alltid

Denna informationsbox visas endast i läge förhandsgranskningen.

Följande kriterium måste vara uppfyllda för att följande fråga ska visas:

Om frågan Hur ofta brukar du skriva ut juvertuber för sintidsbehandling? innehåller något av dessa svarsalternativ

- Mer sällan
- Någon gång i månaden
- Varje vecka

50) Hur ofta rekommenderar du följande uppföljningsrutiner av sintidsbehandling? (ange ett alternativ per rad)

	Aldrig/nästan aldrig	Mindre ofta	Ganska ofta	Alltid/nästan alltid
Kontroll av celltalet vid första provmjölkningen efter kalvning	☐	☐	☐	☐
CMT-undersökning efter kalvning	☐	☐	☐	☐
Mjölkprov för bakteriologisk undersökning efter kalvning	☐	☐	☐	☐
Annat, ange vad i raden nedan	☐	☐	☐	☐

Denna informationsbox visas endast i läge förhandsgranskningen.

Följande kriterium måste vara uppfyllda för att följande fråga ska visas:

Om frågan Hur ofta brukar du skriva ut juvertuber för sintidsbehandling? innehåller något av dessa svarsalternativ

- Mer sällan
- Någon gång i månaden
- Varje vecka

52) Hur anser du att följande aspekter på djurens hälsa och produktion under början av kommande laktation påverkas av sintidsbehandling? (ange ett alternativ per rad)

	Försämras	Ingen effekt	Förbättras	Vet ej
Kons juverhälsa	☐	☐	☐	☐
Kalvens hälsa	☐	☐	☐	☐
Kons mjölkproduktion	☐	☐	☐	☐
Kons livslängd	☐	☐	☐	

FRÅGOR OM BEHANDLING MED SPENFÖRSLUTARE

Definition: Behandling med spenförslutare innebär att juvertuber med vismutsubnitrat (inte antibiotika) sprutas in i alla juverdelar via spenkanalen efter sista mjölkningen under sinläggningen dvs precis innan sinperioden tar vid.

54) Hur ofta brukar du skriva ut spenförslutare?

☐ Varje vecka

☐ Någon gång i månaden

☐ Mer sällan

☐ Aldrig

Denna informationsbox visas endast i läge förhandsgranskningen.

Följande kriterium måste vara uppfyllda för att följande fråga ska visas:

Om frågan Hur ofta brukar du skriva ut spenförslutare? innehåller något av dessa svarsalternativ

- Mer sällan
- Någon gång i månaden
- Varje vecka

55) Brukar du använda dig av bakteriologisk diagnostik innan du ordinerar spenförslutare?

☐ Sällan/aldrig

☐ Mindre ofta

☐ Ganska ofta

☐ Nästan alltid/alltid

Denna informationsbox visas endast i läge förhandsgranskningen.

Följande kriterium måste vara uppfyllda för att följande fråga ska visas:

Om frågan Brukar du använda dig av bakteriologisk diagnostik innan du ordinerar spenförslutare? innehåller något av dessa svarsalternativ

- Nästan alltid/alltid
- Ganska ofta
- Mindre ofta

56) Var sker den bakteriologiska diagnostiken?

☐ Egen odling
☐ På ackrediterat labb
☐ Annat, ange vad

Denna informationsbox visas endast i läge förhandsgranskningen.

Följande kriterium måste vara uppfyllda för att följande fråga ska visas:

Om frågan Hur ofta brukar du skriva ut spenförslutare? innehåller något av dessa svarsalternativ

- Mer sällan
- Någon gång i månaden
- Varje vecka

57) Hur ofta rekommenderar du behandling med spenförslutare till ALLA eller VISSA kor i en besättning? (ett svar per rad)

	Aldrig/nästan aldrig	Mindre ofta	Ganska ofta	Alltid/nästan alltid
Alla kor	○	○	○	○
Vissa kor	○	○	○	○

Denna informationsbox visas endast i läge förhandsgranskningen.

Följande kriterium måste vara uppfyllda för att följande fråga ska visas:

Om frågan Alla kor innehåller något av dessa svarsalternativ

- Alltid/nästan alltid
- Ganska ofta
- Mindre ofta

58) Varför väljer du ibland/alltid att behandla alla kor i en besättning?

☐ Besättningen har/har haft problem med E coli eller Klebsiella-infektioner
☐ Annat, ange vad

Denna informationsbox visas endast i läge förhandsgranskningen.

Följande kriterium måste vara uppfyllda för att följande fråga ska visas:

Om frågan Vissa kor innehåller något av dessa svarsalternativ

- Alltid/nästan alltid
- Ganska ofta
- Mindre ofta

59) Vilken/vilka av följande faktorer påverkar ditt val av kor om du skall behandla vissa kor i besättningen? (flera alternativ möjliga)

☐ Att kon inte haft klinisk (synlig) mastit under laktationen
☐ Kons celltal vid sista provmjölkningen innan sinläggning (ange celltalsgräns i fältet nedan t ex under 200 000/ml)
☐ Kons juverhälsoklass (JHKL) vid sista provmjölkningen innan sinläggning (ange gräns för JHKL i fältet nedan t ex under JHKL3)
☐ Ingen CMT-reaktion i någon juverdel i samband med sinläggningen
☐ Inget bakteriefynd vid odling (ange vilka bakterier som leder till sintidsbehandling i fältet nedan)
☐ Annat, ange vad

Denna informationsbox visas endast i läge förhandsgranskningen.

Följande kriterium måste vara uppfyllda för att följande fråga ska visas:

Om frågan Vissa kor innehåller något av dessa svarsalternativ

- Alltid/nästan alltid
- Ganska ofta
- Mindre ofta

Denna informationsbox visas endast i läge förhandsgranskningen.

Följande kriterium måste vara uppfyllda för att följande fråga ska visas:

Om frågan Hur ofta brukar du skriva ut spenförslutare? innehåller något av dessa svarsalternativ

- Mer sällan
- Någon gång i månaden
- Varje vecka

61) Rekommenderar du alltid att alla fyra juverdelarna behandlas?

○ Nej
○ Ja

Denna informationsbox visas endast i läge förhandsgranskningen.

Följande kriterium måste vara uppfyllda för att följande fråga ska visas:

Om frågan Rekommenderar du alltid att alla fyra juverdelarna behandlas? innehåller något av dessa svarsalternativ

- Nej

62) Vilka juverdelar rekommenderar du ska behandlas?

☐ Endast de som mjölkas vid sinläggningen
☐ Endast de som har lågt CMT
☐ Annat, ange vilka

Denna informationsbox visas endast i läge förhandsgranskningen.

Följande kriterium måste vara uppfyllda för att följande fråga ska visas:

Om frågan Hur ofta brukar du skriva ut spenförslutare? innehåller något av dessa svarsalternativ

- Mer sällan
- Någon gång i månaden
- Varje vecka

63) Brukar du ge råd om hur själva behandlingen bör genomföras?

- ☐ Nej
- ☐ Ja

Denna informationsbox visas endast i läge förhandsgranskningen.

Följande kriterium måste vara uppfyllda för att följande fråga ska visas:

Om frågan Brukar du ge råd om hur själva behandlingen bör genomföras? innehåller något av dessa svarsalternativ

- Nej

64) Varför brukar du inte ge råd om hur själva behandlingen bör genomföras?

- ☐ Anser mig ha för liten kunskap
- ☐ Tidsbrist
- ☐ Upplever inte att det efterfrågas
- ☐ Annat, ange vad

Denna informationsbox visas endast i läge förhandsgranskningen.

Följande kriterium måste vara uppfyllda för att följande fråga ska visas:

Om frågan Brukar du ge råd om hur själva behandlingen bör genomföras? innehåller något av dessa svarsalternativ

- Ja

65) Vilket/vilka av följande moment tycker du bör ingå i en god rutin vid behandling med spenförslutare? (flera alternativ möjliga)

- ☐ Tvätta händerna innan behandling
- ☐ Använda rena handskar
- ☐ Torka av spenarna med papper
- ☐ Torka av spenarna med fuktad engångsduk
- ☐ Torka rent spenspetsen med bifogad servett
- ☐ Torka rent spenspetsen med bomull fuktad med alkohol
- ☐ Använda lång spets (dvs ta bort hela korken) på juvertuben
- ☐ Använda kort spets (dvs endast ta bort yttre änden av korken) på juvertuben

☐ Massera spenen/juverdelen efter att innehållet i juvertuben sprutats in i spenen
☐ Annat, ange vad

Denna informationsbox visas endast i läge förhandsgranskningen.

Följande kriterium måste vara uppfyllda för att följande fråga ska visas:

Om frågan Hur ofta brukar du skriva ut spenförslutare? innehåller något av dessa svarsalternativ

- Mer sällan
- Någon gång i månaden
- Varje vecka

66) Finns det några risker eller svårigheter med behandling med spenförslutare?

- Nej
- Ja, ange vilka

Denna informationsbox visas endast i läge förhandsgranskningen.

Följande kriterium måste vara uppfyllda för att följande fråga ska visas:

Om frågan Hur ofta brukar du skriva ut spenförslutare? innehåller något av dessa svarsalternativ

- Mer sällan
- Någon gång i månaden
- Varje vecka

67) Brukar du rekommendera att spenförslutare används i kombination med sintidsantibiotika?

- Nej
- Ja, ibland
- Ja, alltid

Denna informationsbox visas endast i läge förhandsgranskningen.

Följande kriterium måste vara uppfyllda för att följande fråga ska visas:

Om frågan Brukar du rekommendera att spenförslutare används i kombination med sintidsantibiotika? innehåller något av dessa svarsalternativ

- Ja, ibland

68) * I vilka sammanhang rekommenderar du att spenförslutare används i kombination med sintidsantibiotika?

Denna informationsbox visas endast i läge förhandsgranskningen.

Följande kriterium måste vara uppfyllda för att följande fråga ska visas:

Om frågan Hur ofta brukar du skriva ut spenförslutare? innehåller något av dessa svarsalternativ

- Mer sällan

- Någon gång i månaden
- Varje vecka

69) Hur ofta rekommenderar du följande uppföljningsrutiner av behandlingens effekt? (ange ett alternativ per rad)

	Aldrig/nästan aldrig	Mindre ofta	Ganska ofta	Alltid/nästan alltid
Kontroll av celltalet vid första provmjölkningen efter kalvning	☐	☐	☐	☐
CMT-undersökning efter kalvning	☐	☐	☐	☐
Mjölkprov för bakteriologisk undersökning efter kalvning	☐	☐	☐	☐
Annat, ange vad på raden nedan	☐	☐	☐	☐

Denna informationsbox visas endast i läge förhandsgranskningen.

Följande kriterium måste vara uppfyllda för att följande fråga ska visas:

Om frågan Hur ofta brukar du skriva ut spenförslutare? innehåller något av dessa svarsalternativ

- Mer sällan
- Någon gång i månaden
- Varje vecka

71) Hur anser du att behandling med spenförslutare påverkar följande aspekter på djurens hälsa och produktion under början av kommande laktation? (ange ett alternativ per rad)

	Försämras	Ingen effekt	Förbättras	Vet ej
Kons juverhälsa	☐	☐	☐	☐
Kalvens hälsa	☐	☐	☐	☐
Kons mjölkproduktion	☐	☐	☐	☐
Kons livslängd	☐	☐	☐	☐

FRÅGOR OM SINPERIODEN

Definition: Sinperioden är tiden mellan sista mjölkningen i en laktation och kalvning.

73) Brukar du ge råd om sinperioden till mjölkproducenter och/eller deras personal?

☐ Nej, sällan/aldrig

☐ Ja, ibland

☐ Ja, ofta

Denna informationsbox visas endast i läge förhandsgranskningen.

Följande kriterium måste vara uppfyllda för att följande fråga ska visas:

Om frågan Brukar du ge råd om sinperioden till mjölkproducenter och/eller deras personal? innehåller något av dessa svarsalternativ

- Nej, sällan/aldrig

74) Varför brukar du inte ge råd om sinperioden? (flera alternativ möjliga)

☐ Anser mig ha för liten kunskap
☐ Tidsbrist
☐ Upplever inte att det efterfrågas
☐ Annat, ange vad

75) Anser du att något/några av följande områden är viktiga under sinperioden? (ett svar per rad)

	Nej	Ja, ganska viktigt	Ja, mycket viktigt	Vet ej
Sinperiodens längd	○	○	○	○
Utfodringen	○	○	○	○
Gruppering av kor	○	○	○	○
Hygienen i kons närmiljö	○	○	○	○
Spendoppning/spensprejning	○	○	○	○
Undersökning av juvret	○	○	○	○

Denna informationsbox visas endast i läge förhandsgranskningen.

Följande kriterium måste vara uppfyllda för att följande fråga ska visas:

Om frågan Sinperiodens längd innehåller något av dessa svarsalternativ

- Ja, mycket viktigt
- Ja, ganska viktigt

76) Hur lång anser du att sinperioden bör vara?

○ Kortare än 6 veckor
○ 6-8 veckor
○ Längre än 8 veckor
○ Annan tid, ange vad

Denna informationsbox visas endast i läge förhandsgranskningen.

Följande kriterium måste vara uppfyllda för att följande fråga ska visas:

Om frågan Utfodringen innehåller något av dessa svarsalternativ

- Ja, mycket viktigt
- Ja, ganska viktigt

77) * Vilka råd brukar du ge om utfodringen?

Denna informationsbox visas endast i läge förhandsgranskningen.

Följande kriterium måste vara uppfyllda för att följande fråga ska visas:

Om frågan Gruppering av kor innehåller något av dessa svarsalternativ

- Ja, mycket viktigt
- Ja, ganska viktigt

78) Hur/var rekommenderar du att sinkorna grupperas?

- [ ] I egen avdelning i samma stall som mjölkkorna
- [ ] I annat stall
- [ ] Med de mjölkande korna
- [ ] Annat, ange vad

Denna informationsbox visas endast i läge förhandsgranskningen.

Följande kriterium måste vara uppfyllda för att följande fråga ska visas:

Om frågan Hygienen i kons närmiljö innehåller något av dessa svarsalternativ

- Ja, mycket viktigt
- Ja, ganska viktigt

Frågor om sinperioden

Definition: Sinperioden är tiden mellan sista mjölkningen i en laktation och kalvning.

79) * Vilka råd brukar du ge om hygien under sinperioden?

Denna informationsbox visas endast i läge förhandsgranskningen.

Följande kriterium måste vara uppfyllda för att följande fråga ska visas:

Om frågan Spendoppning/spensprejning innehåller något av dessa svarsalternativ

- Ja, mycket viktigt
- Ja, ganska viktigt

80) När rekommenderar du att korna spendoppas/-sprejas under sinperioden?

- [ ] Vid fasta tider under dygnet
- [ ] Under viss period av sinperioden
- [ ] Ange när under dygnet samt under vilka perioder här:

Denna informationsbox visas endast i läge förhandsgranskningen.

Följande kriterium måste vara uppfyllda för att följande fråga ska visas:

Om frågan Undersökning av juvret innehåller något av dessa svarsalternativ

- Ja, mycket viktigt
- Ja, ganska viktigt

81) Vilka juverundersökningar rekommenderar du under sinperioden? (flera alternativ möjliga)

- [ ] Titta på juvret

☐ Känna på juvret
☐ Göra CMT-undersökning
☐ Annat, ange vad

82) Anser du att sinperioden är viktig för följande aspekter på djurens hälsa och produktion under början av kommande laktation? (ange ett alternativ per rad)

	Nej	Ja, ganska viktig	Ja, mycket viktig	Vet ej
Kons juverhälsa	○	○	○	○
Kalvens hälsa	○	○	○	○
Kons mjölkproduktion	○	○	○	○
Kons fruktsamhet	○	○	○	○
Förekomst av kalvningsförlamning	○	○	○	○
Förekomst av foderleda hos korna	○	○	○	○

FRÅGOR OM BEHOV AV INFORMATION

84) Skulle du vilja lära dig mer om sinläggning och sinperioden?

○ Nej

○ Ja

Denna informationsbox visas endast i läge förhandsgranskningen.

Följande kriterium måste vara uppfyllda för att följande fråga ska visas:

Om frågan Skulle du vilja lära dig mer om sinläggning och sinperioden? innehåller något av dessa svarsalternativ

- Ja

85) Hur vill du helst få tillgång till mer kunskap om sinläggning och sinperiod?

☐ Via kollegor
☐ Via kurser och konferenser
☐ Via facktidskrifter
☐ Via epost-utskick
☐ Via webbverktyg t ex hemsidor, filmer
☐ Annat, ange vad

86) Skulle du vilja lära dig mer om sintidsbehandling och behandling med spenförslutare?

○ Nej

○ Ja

Denna informationsbox visas endast i läge förhandsgranskningen.

Följande kriterium måste vara uppfyllda för att följande fråga ska visas:

Om frågan Skulle du vilja lära dig mer om sintidsbehandling och behandling med spenförslutare? innehåller något av dessa svarsalternativ

- Ja

87) Hur vill du helst få tillgång till mer kunskap om sintidsbehandling och behandling med spenförslutare?

☐ Via kollegor
☐ Via kurser och konferenser
☐ Via facktidskrifter
☐ Via epost-utskick
☐ Via webbverktyg t ex hemsidor, filmer
☐ Annat, ange vad

Supplementary Table S1. Significant associations between herd variables and responses to questions about routines for dry cow therapy with antibiotics (DCT) of dairy cows given by farmers (n = 338) participating in a web-based questionnaire as analysed using univariable logistic or multinominal logistic regression models

Do you use DCT?	Yes N (%)	No N (%)	OR[1]	P-value[3]
Milk production, kg ECM[2]/cow/year				R^2 = 0.04
<9,000	26 (65)	14 (35)	Ref.	
9,000-11,000	166 (85)	29 (15)	0.32	0.004
>11,000	91 (90)	10 (10)	0.20	0.001
Number of cows/herd				R^2 = 0.03
<53	63 (76)	20 (24)	3.54	0.007
53-77	68 (82(	15 (18)	2.46	0.07
78-137	73 (86)	12 (14)	1.83	0.23
≥138	78 (92)	7 (8)	Ref.	

[1] OR = odds ratio; Ref. = referent. [2] ECM = energy-corrected milk. [3]R^2 = coefficient of variation (the proportion of the variation in the dependent variable that is predictable from the independent variable).

DCT used due to udder health problems?	Yes N (%)	Not given as reason N (%)	OR[1]	P-value[2]
Bulk milk SCC, cells/ml				R^2 = 0.04
<200,000	78 (42)	108 (58)	Ref.	
≥200,000	64 (67)	31 (33)	2.85	<0.001

[1] OR = odds ratio; Ref. = referent. [2] R^2 = coefficient of variation (the proportion of the variation in the dependent variable that is predictable from the independent variable).

DCT used due to recommendation from advisor?	Yes N (%)	Not given as reason N (%)	OR[1]	P-value[2]
Milk production, kg ECM/cow/year				R^2 = 0.03
<9,000	3 (12)	23 (88)	Ref.	
9,000-11,000	61 (37)	104 (63)	4.50	0.02
>11,000	42 (46)	49 (54)	6.57	0.004

[1] OR = odds ratio; Ref. = referent. [2] R^2 = coefficient of variation (the proportion of the variation in the dependent variable that is predictable from the independent variable).

How many cows were treated last year (if treating some of the cows)?	<1 of 4 cows N (%)	1 of 4 cows N (%)	≥2 of 4 cows	RRR[1]	P-value[3]
Milk production, kg ECM[2]/cow/year					R^2 = 0.04
<9,000	21 (84)	3 (12)		0.10	<0.001
9,000-11,000	84 (53)	57 (36)		0.47	0.01
>11,000	30 (34)	43 (48)		Ref.	
<9,000	21 (84)		1 (4)	0.09	0.02
9,000-11,000	84 (53)		17 (11)	0.38	0.02
>11,000	30 (34)		16 (18)	Ref.	
<9,000		3 (12)	1 (4)	0.90	0.93
9,000-11,000		57 (36)	17 (11)	0.80	0.58
>11,000		43 (48)	16 (18)	Ref.	
Bulk milk SCC, cells/ml					R^2 = 0.01

<200,000	98 (55)	63 (35)		Ref.	
≥200,000	37 (40)	39 (42)		1.64	0.08
<200,000	98 (55)		17 (10)	Ref.	
≥200,000	37 (40)		17 (18)	2.65	0.01
<200,000		63 (35)	17 (10)	Ref.	
≥200,000		39 (42)	17 (18)	1.61	0.23
Milking system					$R^2 = 0.04$
AMS	50 (42)	53 (45)		2.62	0.003
Tie-stall	52 (65)	21 (26)		Ref.	
Parlour	33 (50)	24 (36)		1.80	0.11
Rotary	0 (0)	4 (67)		-	-
Combinations	0 (0)	2 (100)		-	-
AMS	50 (42)		16 (13)	2.38	0.08
Tie-stall	52 (65)		7 (9)	Ref.	
Parlour	33 (50)		9 (14)	2.03	0.20
Rotary	0 (0)		2 (33)	-	-
Combinations	0 (0)		0 (0)	-	-
AMS		53 (45)	16 (13)	0.91	0.85
Tie-stall		21 (26)	7 (9)	Ref.	
Parlour		24 (36)	9 (14)	1.12	0.84
Rotary		4 (67)	2 (33)	1.50	0.68
Combinations		2 (100)	0 (0)	-	-
Number of cows/herd					$R^2 = 0.03$
<53	42 (68)	15 (24)		Ref.	
53-77	31 (47)	26 (39)		2.35	0.03
78-137	34 (49)	28 (41)		2.31	0.03
≥138	26 (35)	35 (47)		3.77	0.001
<53	42 (68)		5 (8)	Ref.	
53-77	31 (47)		9 (14)	2.44	0.14
78-137	34 (49)		7 (10)	1.73	0.38
≥138	26 (35)		13 (18)	4.20	0.01
<53		15 (24)	5 (8)	1.33	0.67
53-77		26 (39)	9 (14)	1.38	0.57
78-137		28 (41)	7 (10)	Ref.	
≥138		35 (47)	13 (18)	1.49	0.46

[1] RRR = relative risk ratio; Ref. = referent; AMS = automatic milking system. [2] ECM = energy-corrected milk. [3] R^2 = coefficient of variation (the proportion of the variation in the dependent variable that is predictable from the independent variable).

Was Benestermycin® used for DCT last year?	Yes N (%)	Not given as answer N (%)	OR[1]	P-value[3]
Region[2]				$R^2 = 0.05$
East Sweden	12 (29)	30 (71)	2.27	0.14
Norrland	6 (15)	34 (85)	Ref.	
Northern Middle Sweden	8 (33)	16 (67)	2.83	0.09
Småland and the islands	22 (39)	35 (61)	3.56	0.02
South Sweden	12 (43)	16 (57)	4.25	0.01

West Sweden	42 (52)	38 (48)	6.26	<0.001
Milk production, kg ECM[4]/cow/year[5]				R^2 = 0.06
<9,000	1 (4)	24 (96)	Ref.	
9,000-11,000	56 (36)	101 (64)	13.3	0.01
>11,000	44 (50)	44 (50)	24.0	0.002

[1] OR = odds ratio; Ref. = referent. [2] In addition, a higher proportion of herds in West Sweden than in East Sweden (RRR = 2.76, P = 0.01) used Benestermycin®. [3] R^2 = coefficient of variation (the proportion of the variation in the dependent variable that is predictable from the independent variable). [4] ECM = energy-corrected milk. [5] In addition, a higher proportion of herds producing >11,000 kg ECM than in herds with 9,000-11,000 kg ECM (RRR = 1.80, P = 0.03) used Benestermycin®.

Was Siccalactin® used for DCT last year?	Yes N (%)	Not given as answer N (%)	OR[1]	P-value[3]
Region[2]				R^2 = 0.08
East Sweden	35 (83)	7 (17)	3.16	0.01
Norrland	38 (95)	2 (5)	12.0	0.001
Northern Middle Sweden	21 (87)	3 (13)	4.43	0.02
Småland and the islands	44 (77)	13 (23)	2.14	0.05
South Sweden	17 (61)	11 (39)	0.98	0.96
West Sweden	49 (61)	31 (39)	Ref.	
Milk production, kg ECM[4]/cow/year				R^2 = 0.04
<9,000	24 (96)	1 (4)	11.8	0.02
9,000-11,000	120 (76)	37 (24)	1.59	0.11
>11,000	59 (67)	29 (33)	Ref.	

[1] OR = odds ratio; Ref. = referent. [2] In addition, a higher proportion of herds in Norrland than in Småland and the islands (RRR = 5.61, P = 0.03) used Siccalactin®. [3] R^2 = coefficient of variation (the proportion of the variation in the dependent variable that is predictable from the independent variable). [4] ECM = energy-corrected milk.

Was washing hands a part of the routine before DCT?	Yes N (%)	Not given as answer N (%)	OR[1]	P-value[2]
Milking system				R^2 = 0.03
AMS[1]	71 (60)	48 (40)	2.77	0.01
Tie-stall	42 (52)	38 (48)	2.07	0.03
Parlour	23 (35)	43 (65)	Ref.	
Rotary	3 (50)	3 (50)	1.87	0.47
Combinations	1 (50)	1 (50)	1.87	0.66

[1] OR = odds ratio; Ref. = referent; AMS = automatic milking system. [2] R^2 = coefficient of variation (the proportion of the variation in the dependent variable that is predictable from the independent variable).

Was using clean gloves a part of the routine at DCT?	Yes N (%)	Not given as answer N (%)	OR[1]	P-value[2]
Milking system				R^2 = 0.03
AMS[1]	38 (32)	81 (68)	0.74	0.32
Tie-stall	31 (39)	49 (61)	Ref.	
Parlour	38 (58)	28 (42)	2.14	0.02
Rotary	3 (50)	3 (50)	1.58	0.59
Combinations	0 (0)	2 (100)	-	-
Milk production, kg ECM[3]/cow/year				R^2 = 0.03
<9,000	4 (16)	21 (84)	Ref.	
9,000-11,000	62 (39)	96 (61)	3.39	0.03

>11,000	44 (49)	45 (51)	5.13	0.005

[1] OR = odds ratio; Ref. = referent; AMS = automatic milking system. [2] R^2 = coefficient of variation (the proportion of the variation in the dependent variable that is predictable from the independent variable). [3] ECM = energy-corrected milk.

Was wiping the teats with paper a part of the routine at DCT?	Yes N (%)	Not given as answer N (%)	OR[1]	P-value[2]
Production type				$R^2 = 0.01$
Conventional	51 (23)	171 (77)	Ref.	
Organic	19 (37)	32 (63)	1.99	0.04

[1] OR = odds ratio; Ref. = referent. [2] R^2 = coefficient of variation (the proportion of the variation in the dependent variable that is predictable from the independent variable).

Was wiping the teats with a moist single-use cloth a part of the routine at DCT?	Yes N (%)	Not given as answer N (%)	OR[1]	P-value[2]
Milking system				$R^2 = 0.05$
AMS[1]	27 (23)	92 (77)	Ref.	
Tie-stall	35 (44)	45 (56)	2.65	0.002
Parlour	34 (52)	32 (48)	3.62	<0.001
Rotary	1 (17)	5 (83)	0.68	0.73
Combinations	0 (0)	2 (100)	1.00	-

[1] OR = odds ratio; Ref. = referent; AMS = automatic milking system. [2] R^2 = coefficient of variation (the proportion of the variation in the dependent variable that is predictable from the independent variable).

Was use of long tip (full insertion) a part of the routine at DCT?	Yes N (%)	Not given as answer N (%)	OR[1]	P-value[3]
Region[2]				$R^2 = 0.08$
East Sweden	35 (83)	7 (17)	2.12	0.14
Norrland	38 (95)	2 (5)	8.07	0.007
Northern Middle Sweden	20 (83)	4 (17)	2.12	0.22
Småland and the islands	40 (70)	17 (30)	Ref.	
South Sweden	27 (93)	2 (7)	5.74	0.03
West Sweden	65 (80)	16 (20)	1.73	0.17

[1] OR = odds ratio; Ref. = referent. [2] In addition, the proportion of herds using long tip was higher in Norrland than in West Sweden (RRR = 4.68, P = 0.05). [3] R^2 = coefficient of variation (the proportion of the variation in the dependent variable that is predictable from the independent variable).

Are there any risks or difficulties with DCT?	Yes N (%)	No N (%)	OR[1]	P-value[2]
Production type				$R^2 = 0.02$
Conventional	55 (25)	166 (75)	Ref.	
Organic	23 (45)	28 (55)	2.48	0.005

[1] OR = odds ratio; Ref. = referent. [2] R^2 = coefficient of variation (the proportion of the variation in the dependent variable that is predictable from the independent variable).

How often do you control the effect of DCC by examining the CSCC[1] at first milk recording after calving?	Always/ almost always N (%)	Quite often N (%)	Less often N (%)	Never/ almost never N (%)	RRR[1]	P-value[2]
Number of cows/herd						$R^2 = 0.04$

<53	47 (75)	7 (11)			Ref.	
53-77	43 (65)	15 (23)			2.34	0.09
78-137	33 (46)	18 (25)			3.66	0.009
≥138	37 (47)	13 (17)			2.42	0.09
<53	47 (75)		5 (8)		2.29	0.34
53-77	43 (65)		2 (3)		Ref.	
78-137	33 (46)		8 (11)		5.21	0.05
≥138	36 (47)		9 (12)		5.37	0.04
<53	47 (75)			4 (6)	Ref.	
53-77	43 (65)			6 (9)	1.64	0.47
78-137	33 (46)			12 (17)	4.27	0.02
≥138	36 (47)			18 (24)	5.87	0.003
<53		7 (11)	5 (8)		5.36	0.08
53-77		15 (23)	2 (3)		Ref.	
78-137		18 (25)	8 (11)		3.33	0.16
≥138		13 (17)	9 (12)		5.19	0.06
<53		7 (11)		4 (6)	1.43	0.65
53-77		15 (23)		6 (9)	Ref.	
78-137		18 (25)		12 (17)	1.67	0.40
≥138		13 (17)		18 (24)	3.46	0.04
<53			5 (8)	4 (6)	0.27	0.21
53-77			2 (3)	6 (9)	Ref.	
78-137			8 (11)	12 (17)	0.50	0.46
≥138			9 (12)	18 (24)	0.67	0.66

[1] RRR = relative risk ratio; Ref. = referent; CSCC = cow somatic cell count. [2] R^2 = coefficient of variation (the proportion of the variation in the dependent variable that is predictable from the independent variable).

How often do you control the effect of DCC by doing CMT[1] after calving?	Always/ almost always N (%)	Quite often N (%)	Less often N (%)	Never/ almost never N (%)	RRR[1]	P-value[2]
Bulk milk SCC						R^2 = 0.04
<200,000	55 (32)	33 (19)			Ref.	
≥200,000	16 (18)	14 (16)			1.46	0.38
<200,000	55 (32)		31 (18)		Ref.	
≥200,000	16 (18)		27 (30)		2.99	0.005
<200,000	55 (32)			53 (31)	Ref.	
≥200,000	16 (18)			33 (37)	2.14	0.04
<200,000		33 (19)	31 (18)		Ref.	
≥200,000		14 (16)	27 (30)		2.05	0.08
<200,000		33 (19)		53 (31)	Ref.	
≥200,000		14 (16)		33 (37)	1.47	0.32
<200,000			31 (18)	53 (31)	Ref.	
≥200,000			27 (30)	33 (37)	0.71	0.33

Milking system						$R^2 = 0.03$
AMS[1]	21 (18)	20 (17)			Ref.	
Tie-stall	24 (32)	15 (20)			0.66	0.35
Parlour	22 (34)	12 (19)			0.57	0.24
Rotary	3 (50)	0 (0)			-	-
Combinations	1 (50)	0 (0)			-	-
AMS	21 (18)		33 (28)		4.32	0.003
Tie-stall	24 (32)		18 (24)		2.06	0.08
Parlour	22 (34)		8 (12)		Ref.	
Rotary	3 (50)		0 (0)		-	-
Combinations	1 (50)		0 (0)		-	-
AMS	21 (18)			43 (27)	2.73	0.04
Tie-stall	24 (32)			18 (24)	Ref.	
Parlour	22 (34)			22 (34)	1.33	0.51
Rotary	3 (50)			3 (50)	1.33	0.74
Combinations	1 (50)			1 (50)	1.33	0.84
AMS		20 (17)	33 (28)		Ref.	
Tie-stall		15 (20)	18 (24)		0.73	0.48
Parlour		12 (19)	8 (12)		0.40	0.09
Rotary		0 (0)	0 (0)		-	-
Combinations		0 (0)	0 (0)		-	-
AMS		20 (17)		43 (27)	Ref.	
Tie-stall		15 (20)		18 (24)	0.56	0.19
Parlour		12 (19)		22 (34)	0.85	0.72
Rotary		0 (0)		3 (50)	-	-
Combinations		0 (0)		1 (50)	-	-
AMS			33 (28)	43 (27)	Ref.	
Tie-stall			18 (24)	18 (24)	0.77	0.51
Parlour			8 (12)	22 (34)	2.11	0.12
Rotary			0 (0)	3 (50)	-	-
Combinations			0 (0)	1 (50)	-	-

[1] RRR = relative risk ratio; Ref. = referent; AMS = automatic milking system; CMT = California mastitis test.
[2] R^2 = coefficient of variation (the proportion of the variation in the dependent variable that is predictable from the independent variable).

Supplementary Table S2. Significant associations between veterinary variables and responses to questions about advice on dry cow therapy with antibiotics of dairy cows given by veterinarians (n = 130) participating in a web-based questionnaire as analysed using univariable logistic or multinominal logistic regression models

How often do you prescribe DCC?	Each week N (%)	A couple of times per month N (%)	Less often N (%)	Never N (%)	RRR[1]	P-value[2]
Region						$R^2 = 0.12$
East Sweden	3 (9)	14 (42)			0.36	0.21
Norrland	4 (17)	11 (48)			0.61	0.52
Northern Middle Sweden	0 (0)	6 (33)			-	-
Småland and the islands	6 (33)	10 (56)			Ref.	
South Sweden	1 (7)	11 (73)			0.15	0.11
West Sweden	6 (26)	12 (52)			0.83	0.80
East Sweden	3 (9)		13 (39)		Ref.	
Norrland	4 (17)		8 (35)		2.17	0.38
Northern Middle Sweden	0 (0)		12 (67)		-	-
Småland and the islands	6 (33)		2 (11)		13.0	0.01
South Sweden	1 (7)		3 (20)		1.44	0.78
West Sweden	6 (26)		4 (17)		6.50	0.04
East Sweden	3 (9)			3 (9)	Ref.	
Norrland	4 (17)			0 (0)	-	-
Northern Middle Sweden	0 (0)			0 (0)	-	-
Småland and the islands	6 (33)			0 (0)	-	-
South Sweden	1 (7)			0 (0)	-	-
West Sweden	6 (26)			1 (4)	6.00	0.19
East Sweden		14 (42)	13 (39)		2.15	0.22
Norrland		11 (48)	8 (35)		2.75	0.14
Northern Middle Sweden		6 (33)	12 (67)		Ref.	
Småland and the islands		10 (56)	2 (11)		10.0	0.01
South Sweden		11 (73)	3 (20)		7.33	0.02
West Sweden		12 (52)	4 (17)		6.00	0.02
East Sweden		14 (42)		3 (9)	Ref.	
Norrland		11 (48)		0 (0)	-	-
Northern Middle Sweden		6 (33)		0 (0)	-	-
Småland and the islands		10 (56)		0 (0)	-	-
South Sweden		11 (73)		0 (0)	-	-
West Sweden		12 (52)		1 (4)	2.57	0.44
East Sweden			13 (39)	3 (9)	Ref.	
Norrland			8 (35)	0 (0)	-	-
Northern Middle Sweden			12 (67)	0 (0)	-	-
Småland and the islands			2 (11)	0 (0)	-	-
South Sweden			3 (20)	0 (0)	-	-
West Sweden			4 (17)	1 (4)	0.92	0.95
Post-graduate training						$R^2 = 0.05$

Yes	17 (22)	42 (55)			Ref.	
No	3 (6)	22 (42)			0.34	0.11
Yes	17 (22)		17 (22)		Ref.	
No	3 (6)		24 (46)		0.12	0.003
Yes	17 (22)			1 (1)	Ref.	
No	3 (6)			3 (6)	0.06	0.03
Yes		42 (55)	17 (22)		Ref.	
No		22 (42)	24 (46)		0.37	0.02
Yes		42 (55)		1 (1)	Ref.	
No		22 (42)		3 (6)	0.17	0.14
Yes			17 (22)	1 (1)	Ref.	
No			24 (46)	3 (6)	0.47	0.53
Number of mastitis cases/month						$R^2 = 0.20$
<1	0 (0)	4 (23)			-	-
1-3	2 (7)	13 (43)			0.13	0.03
4-8	3 (8)	21 (52)			0.12	0.01
9-15	7 (26)	19 (70)			0.32	0.10
>15	8 (50)	7 (44)			Ref.	
<1	0 (0)		11 (65)		-	-
1-3	2 (7)		15 (50)		0.02	0.002
4-8	3 (8)		15 (37)		0.03	0.004
9-15	7 (26)		1 (4)		Ref.	
>15	8 (50)		0 (0)		-	-
<1	0 (0)			2 (12)	-	-
1-3	2 (7)			0 (0)	-	-
4-8	3 (8)			1 (3)	0.37	0.53
9-15	7 (26)			0 (0)	-	-
>15	8 (50)			1 (6)	Ref.	
<1		4 (23)	11 (65)		0.02	0.001
1-3		13 (43)	15 (50)		0.05	0.005
4-8		21 (52)	15 (37)		0.07	0.02
9-15		19 (70)	1 (4)		Ref.	
>15		7 (44)	0 (0)		-	-
<1		4 (23)		2 (12)	Ref.	
1-3		13 (43)		0 (0)	-	-
4-8		21 (52)		1 (3)	10.5	0.08
9-15		19 (70)		0 (0)	-	-
>15		7 (44)		1 (6)	3.50	0.36
<1			11 (65)	2 (12)	Ref.	
1-3			15 (50)	0 (0)	-	-
4-8			15 (37)	1 (3)	2.73	0.44
9-15			1 (4)	0 (0)	-	-
>15			0 (0)	1 (6)	-	-

[1] RRR = relative risk ratio; Ref. = referent; AMS = automatic milking system. [2] R^2 = coefficient of variation (the proportion of the variation in the dependent variable that is predictable from the independent variable).

Do you do your own culture for bacteriology of milk samples before prescribing DCT?	Yes N (%)	No N (%)	OR[1]	P-value[2]
Region				R^2 = 0.11
East Sweden	11 (44)	14 (56)	0.14	0.01
Norrland	16 (84)	3 (16)	Ref.	
North Middle Sweden	5 (31)	11 (69)	0.08	0.003
Småland and the islands	7 (58)	5 (42)	0.26	0.12
South Sweden	3 (33)	6 (67)	0.09	0.01
West Sweden	10 (62)	6 (38)	0.31	0.15

[1] OR = odds ratio; Ref. = referent. [2] R^2 = coefficient of variation (the proportion of the variation in the dependent variable that is predictable from the independent variable).

Do you use an accredited laboratory for bacteriology of milk samples before prescribing DCT?	Yes N (%)	No N (%)	OR[1]	P-value[3]
Number of mastitis cases/month[2]				R^2 = 0.12
<1	7 (54)	6 (46)	2.04	0.39
1-3	22 (88)	3 (12)	12.8	0.004
4-8	25 (81)	6 (19)	7.29	0.01
9-15	10 (59)	7 (41)	2.50	0.25
>15	4 (36)	7 (64)	Ref.	

[1] OR = odds ratio; Ref. = referent. [2] In addition, a larger proportion of veterinarians treating 1-3 cases/month than of those treating <1 case/month (OR = 6.59, P = 0.03) or 9-15 cases/month (OR = 5.13, P = 0.04) send samples to an accredited laboratory. [3] R^2 = coefficient of variation (the proportion of the variation in the dependent variable that is predictable from the independent variable).

Which DCT product do you usually prescribe?	Siccalactin® N (%)	Benestermycin® N (%)	OR[1]	P-value[3]
Region[2]				R^2 = 0.13
East Sweden	19 (73)	7 (27)	0.26	0.04
Norrland	20 (87)	3 (13)	0.10	0.004
North Middle Sweden	15 (94)	1 (6)	0.05	0.007
Småland and the islands	7 (41)	10 (59)	Ref.	
South Sweden	7 (58)	5 (42)	0.50	0.36
West Sweden	10 (53)	9 (47)	0.63	0.49
Post-graduate training				R^2 = 0.07
Yes	41 (59)	29 (41)	4.24	0.004
No	36 (86)	6 (14)	Ref.	
Number of mastitis cases/month[4]				R^2 = 0.11
<1	14 (93)	1 (7)	Ref.	
1-3	20 (71)	8 (29)	1.72	0.12
4-8	27 (79)	7 (21)	1.29	0.25
9-15	13 (52)	12 (48)	2.26	0.02
>15	4 (36)	7 (64)	3.20	0.008

[1] OR = odds ratio; Ref. = referent. [2] In addition, a larger proportion of veterinarians in South Sweden (OR = 10.7, P = 0.046) or West Sweden (OR = 13.5, P = 0.002) than in North Middle Sweden, and a larger proportion of veterinarians in West Sweden than in Norrland (OR = 6.00, P = 0.02) answered Benestermycin®. [3] R^2 = coefficient of variation (the proportion of the variation in the dependent variable

that is predictable from the independent variable). [4] In addition, a larger proportion of veterinarians treating 9-15 cases/month (OR = 3.56, P = 0.03) or >15 cases/month (OR = 6.75, P = 0.01) than of those treating 4-8 cases/month answered Benestermycin®.

How often do you recommend DCT to some (rather than all) cows in a herd?	Always/ almost always N (%)	Quite often N (%)	Less often N (%)	RRR[1]	P-value[2]
Post-graduate training					$R^2 = 0.08$
Yes	45 (59)	28 (37)		Ref.	
No	13 (27)	24 (49)		2.97	0.01
Yes	45 (59)		3 (4)	Ref.	
No	13 (27)		12 (24)	13.9	<0.001
Yes		28 (37)	3 (4)	Ref.	
No		24 (49)	12 (24)	4.67	0.03

[1] RRR = relative risk ratio; Ref. = referent. [2] R^2 = coefficient of variation (the proportion of the variation in the dependent variable that is predictable from the independent variable).

Do the CMT-reaction affect the selection of cows for DCT?	Yes N (%)	No N (%)	OR[1]	P-value[2]
Post-graduate training				$R^2 = 0.05$
Yes	22 (30)	51 (70)	Ref.	
No	28 (57)	21 (43)	3.09	0.003

[1] OR = odds ratio; Ref. = referent. [2] R^2 = coefficient of variation (the proportion of the variation in the dependent variable that is predictable from the independent variable).

Do you give advice on how to perform the DCT?	Yes, often N (%)	Yes, sometimes N (%)	No N (%)	RRR[1]	P-value[2]
Post-graduate training					$R^2 = 0.06$
Yes	9 (12)	48 (64)		Ref.	
No	3 (6)	18 (37)		0.89	0.87
Yes	9 (12)		18 (24)	Ref.	
No	3 (6)		28 (57)	0.21	0.04
Yes		48 (64)	18 (24)	Ref.	
No		18 (37)	28 (57)	0.24	0.001
Number of mastitis cases/month					$R^2 = 0.07$
<1	1 (7)	6 (40)		0.23	0.24
1-3	2 (7)	12 (40)		0.23	0.13
4-8	1 (3)	24 (61)		0.06	0.02
9-15	3 (12)	17 (65)		0.25	0.10
>15	5 (33)	7 (47)		Ref.	
<1	1 (7)		8 (53)	0.07	0.04
1-3	2 (7)		16 (53)	0.07	0.01
4-8	1 (3)		14 (36)	0.04	0.01
9-15	3 (12)		6 (23)	0.30	0.34
>15	5 (33)		3 (20)	Ref.	
<1		6 (40)	8 (53)	0.26	0.07

1-3	12 (40)	16 (53)	0.26	0.03
4-8	24 (61)	14 (36)	0.60	0.39
9-15	17 (65)	6 (23)	Ref.	
>15	7 (47)	3 (20)	0.82	0.82

[1] RRR = relative risk ratio; Ref. = referent. [2] R^2 = coefficient of variation (the proportion of the variation in the dependent variable that is predictable from the independent variable).

Is washing hands included in a good routine before DCT?	Yes N (%)	Not given as answer N (%)	OR[1]	P-value[2]
Gender				R^2 = 0.07
Female	47 (80)	12 (20)	Ref.	
Male	9 (47)	10 (53)	0.22	0.009
Number of mastitis cases/month				R^2 = 0.11
<1	6 (86)	1 (14)	0.46	0.61
1-3	13 (93)	1 (7)	Ref.	
4-8	20 (80)	5 (20)	0.31	0.31
9-15	11 (55)	9 (45)	0.09	0.04
>15	6 (50)	6 (50)	0.08	0.03

[1] OR = odds ratio; Ref. = referent. [2] R^2 = coefficient of variation (the proportion of the variation in the dependent variable that is predictable from the independent variable).

Is wiping the teats with paper included in a good routine before DCT?	Yes N (%)	Not given as answer N (%)	OR[1]	P-value[2]
Country of degree				R^2 = 0.14
Sweden	13 (20)	51 (80)	0.04	0.005
Denmark/Norway/Finland	1 (14)	6 (86)	0.03	0.02
Other European countries	6 (86)	1 (14)	Ref.	

[1] OR = odds ratio; Ref. = referent. [2] R^2 = coefficient of variation (the proportion of the variation in the dependent variable that is predictable from the independent variable).

Is using a long tube tip (full insertion) included in a good routine before DCT?	Yes N (%)	Not given as answer N (%)	OR[1]	P-value[2]
Post-graduate training				R^2 = 0.08
Yes	20 (35)	37 (65)	Ref.	
No	15 (71)	6 (29)	4.62	0.006

[1] OR = odds ratio; Ref. = referent. [2] R^2 = coefficient of variation (the proportion of the variation in the dependent variable that is predictable from the independent variable).

Do you know about the legislation about DCT?	Yes N (%)	No N (%)	OR[1]	P-value[2]
Post-graduate training				R^2 = 0.13
Yes	63 (83)	13 (17)	Ref.	
No	23 (44)	29 (56)	6.11	<0.001

[1] OR = odds ratio; Ref. = referent. [2] R^2 = coefficient of variation (the proportion of the variation in the dependent variable that is predictable from the independent variable).

How often do you follow the legislation on DCT?	Always/ almost always N (%)	Quite often N (%)	Less often/ Never N (%)	RRR[1]	P-value[3]
Year of degree[2]					R^2 = 0.10
1977-1991	18 (72)	6 (24)		Ref.	

1992-2001	14 (54)	11 (42)		2.36	0.17
2002-2008	9 (39)	11 (48)		3.67	0.05
2009-2014	14 (56)	3 (12)		0.64	0.58
2015-2020	8 (30)	11 (41)		4.12	0.03
1977-1991	18 (72)		1 (4)	Ref.	
1992-2001	14 (54)		1 (4)	1.29	0.86
2002-2008	9 (39)		3 (13)	6.0	0.14
2009-2014	14 (56)		8 (32)	10.3	0.04
2015-2020	8 (30)		8 (30)	18.0	0.01
1977-1991		6 (24)	1 (4)	0.06	0.03
1992-2001		11 (42)	1 (4)	0.03	0.007
2002-2008		11 (48)	3 (13)	0.10	0.02
2009-2014		3 (12)	8 (32)	Ref.	
2015-2020		11 (41)	8 (30)	0.27	0.11
Region[4]					$R^2 = 0.08$
East Sweden	15 (50)	12 (40)		4.80	0.07
Norrland	14 (61)	8 (35)		3.43	0.16
North Middle Sweden	9 (5)	7 (39)		4.67	0.09
Småland and the islands	5 (28)	6 (33)		7.20	0.04
South Sweden	12 (80)	2 (13)		Ref.	
West Sweden	8 (36)	7 (32)		5.25	0.07
East Sweden	15 (50)		3 (10)	0.14	0.02
Norrland	14 (61)		1 (4)	0.05	0.01
North Middle Sweden	9 (5)		2 (11)	0.16	0.06
Småland and the islands	5 (28)		7 (39)	Ref.	
South Sweden	12 (80)		1 (7)	0.06	0.02
West Sweden	8 (36)		7 (32)	0.63	0.55
East Sweden		12 (40)	3 (10)	0.21	0.07
Norrland		8 (35)	1 (4)	0.11	0.06
North Middle Sweden		7 (39)	2 (11)	0.24	0.15
Småland and the islands		6 (33)	7 (39)	Ref.	
South Sweden		2 (13)	1 (7)	0.43	0.53
West Sweden		7 (32)	7 (32)	0.86	0.84
Gender					$R^2 = 0.02$
Female	52 (52)	35 (35)		Ref.	
Male	11 (41)	7 (36)		0.94	0.92
Female	52 (52)		12 (12)	Ref.	
Male	11 (41)		9 (33)	3.54	0.02
Female		35 (35)	12 (12)	Ref.	
Male		7 (36)	9 (33)	3.75	0.03
Number of mastitis cases/month					$R^2 = 0.08$
<1	9 (60)	5 (33)		0.93	0.92
1-3	15 (50)	12 (40)		1.33	0.66
4-8	22 (56)	15 (38)		1.14	0.84
9-15	10 (37)	6 (22)		Ref.	

>15	7 (47)	4 (27)		0.95	0.95
<1	9 (60)		1 (7)	0.10	0.05
1-3	15 (50)		3 (10)	0.18	0.03
4-8	22 (56)		2 (5)	0.08	0.004
9-15	10 (37)		11 (41)	Ref.	
>15	7 (47)		4 (27)	0.52	0.39
<1		5 (33)	1 (7)	0.11	0.07
1-3		12 (40)	3 (10)	0.14	0.02
4-8		15 (38)	2 (5)	0.07	0.004
9-15		6 (22)	11 (41)	Ref.	
>15		4 (27)	4 (27)	0.54	0.49
Number of years in cattle practice					$R^2 = 0.10$
<5	9 (32)	10 (36)		5.93	0.02
5-9	12 (55)	4 (18)		1.78	0.50
10-14	7 (37)	9 (47)		6.86	0.02
15-19	13 (65)	6 (20)		2.46	0.26
20-24	6 (35)	10 (59)		8.89	0.007
≥25	16 (80)	3 (15)		Ref.	
<5	9 (32)		9 (32)	Ref.	
5-9	12 (55)		6 (27)	0.50	0.31
10-14	7 (37)		3 (15)	0.43	0.32
15-19	13 (65)		1 (5)	0.08	0.02
20-24	6 (35)		1 (6)	0.17	0.13
≥25	16 (80)		1 (5)	0.06	0.01
<5		10 (36)	9 (32)	9.0	0.06
5-9		4 (18)	6 (27)	15.0	0.03
10-14		9 (47)	3 (15)	3.33	0.33
15-19		6 (20)	1 (5)	1.67	0.73
20-24		10 (59)	1 (6)	Ref.	
≥25		3 (15)	1 (5)	3.33	0.44

[1] RRR = relative risk ratio; Ref. = referent. [2] In addition, a larger proportion of veterinarians with a degree from 2002-2008 (RRR = 1.74, P = 0.03) or 2015-2020 (RRR = 1.86, P = 0.02) followed the legislation quite often compared with those with a degree from 2009-2014 who more often always/almost always followed the legislation. A larger proportion of those with a degree from 2015-2020 (RRR = 2.64, P = 0.02) less often or never/almost never followed the legislation than those with a degree from 1992-2001 who more often always/almost always followed the legislation. [3] R^2 = coefficient of variation (the proportion of the variation in the dependent variable that is predictable from the independent variable). [4] In addition, a larger proportion of veterinarians in West Sweden stated less often or never/almost never than those in Norrland (RRR = 0.08, P = 0.03) or South Sweden (RRR = 0.09, P = 0.04) who more often stated always/almost always.

How often do you recommend follow-up of the DCT effect by examining the CSCC[1] after calving?	Always/ almost always N (%)	Quite often N (%)	Less often N (%)	Never/ almost never N (%)	RRR[1]	P-value[2]
Year of degree						$R^2 = 0.10$
1977-1991	14 (58)	6 (25)			Ref.	
1992-2001	11 (46)	11 (46)			2.33	0.19
2002-2008	5 (22)	9 (39)			4.20	0.05
2009-2014	8 (35)	5 (22)			1.46	0.62

2015-2020	6 (22)	7 (26)			2.72	0.18
1977-1991	14 (58)		1 (4)		Ref.	
1992-2001	11 (46)		1 (4)		1.27	0.87
2002-2008	5 (22)		5 (22)		14.0	0.03
2009-2014	8 (35)		3 (13)		5.25	0.18
2015-2020	6 (22)		5 (19)		11.7	0.04
1977-1991	14 (58)			3 (13)	0.14	0.02
1992-2001	11 (46)			1 (4)	0.06	0.02
2002-2008	5 (22)			4 (17)	0.53	0.46
2009-2014	8 (35)			7 (30)	0.58	0.47
2015-2020	6 (22)			9 (33)	Ref.	
1977-1991		6 (25)	1 (4)		1.83	0.69
1992-2001		11 (46)	1 (4)		Ref.	
2002-2008		9 (39)	5 (22)		6.11	0.13
2009-2014		5 (22)	3 (13)		6.60	0.14
2015-2020		7 (26)	5 (19)		7.86	0.09
1977-1991		6 (25)		3 (13)	5.50	0.18
1992-2001		11 (46)		1 (4)	Ref.	
2002-2008		9 (39)		4 (17)	4.89	0.19
2009-2014		5 (22)		7 (30)	15.4	0.02
2015-2020		7 (26)		9 (33)	14.1	0.02
1977-1991			1 (4)	3 (13)	1.67	0.69
1992-2001			1 (4)	1 (4)	0.56	0.70
2002-2008			5 (22)	4 (17)	0.44	0.35
2009-2014			3 (13)	7 (30)	1.30	0.77
2015-2020			5 (19)	9 (33)	Ref.	
Number of years in cattle practice						R^2 = 0.08
<5	6 (21)	7 (25)			3.50	0.12
5-9	6 (30)	5 (25)			2.50	0.27
10-14	6 (32)	7 (37)			3.50	0.12
15-19	6 (32)	8 (42)			4.00	0.08
20-24	8 (50)	7 (44)			2.62	0.21
≥25	12 (63)	4 (21)			Ref.	
<5	6 (21)		5 (18)		10.0	0.06
5-9	6 (30)		2 (15)		6.00	0.15
10-14	6 (32)		4 (21)		8.00	0.09
15-19	6 (32)		1 (5)		2.00	0.64
20-24	8 (50)		1 (6)		1.50	0.79
≥25	12 (63)		1 (5)		Ref.	
<5	6 (21)			10 (36)	10.0	0.01
5-9	6 (30)			6 (30)	6.00	0.06
10-14	6 (32)			2 (10)	2.00	0.54
15-19	6 (32)			4 (21)	4.00	0.17
20-24	8 (50)			0 (0)	-	-
≥25	12 (63)			2 (11)	Ref.	
<5		7 (25)	5 (18)		Ref.	

5-9		5 (25)	2 (15)		0.84	0.85
10-14		7 (37)	4 (21)		0.80	0.80
15-19		8 (42)	1 (5)		0.17	0.15
20-24		7 (44)	1 (6)		0.20	0.19
≥25		4 (21)	1 (5)		0.35	0.40
<5		7 (25)		10 (36)	Ref.	
5-9		5 (25)		6 (30)	0.84	0.09
10-14		7 (37)		2 (10)	0.20	0.18
15-19		8 (42)		4 (21)	0.35	0.99
20-24		7 (44)		0 (0)	-	-
≥25		4 (21)		2 (11)	0.35	0.29
<5			5 (18)	10 (36)	4.00	0.18
5-9			2 (15)	6 (30)	4.00	0.22
10-14			4 (21)	2 (10)	Ref.	
15-19			1 (5)	4 (21)	8.00	0.14
20-24			1 (6)	0 (0)	-	-
≥25			1 (5)	2 (11)	4.00	0.36

[1] RRR = relative risk ratio; Ref. = referent; CSCC = cow somatic cell count. [2] R^2 = coefficient of variation (the proportion of the variation in the dependent variable that is predictable from the independent variable).

How often do you recommend follow-up of the DCT effect by CMT[1] after calving?	Always/ almost always N (%)	Quite often N (%)	Less often N (%)	Never/ almost never N (%)	RRR[1]	P-value[2]
Year of degree						R^2 = 0.11
1977-1991	13 (52)	8 (32)			Ref.	
1992-2001	10 (40)	11 (44)			1.79	0.35
2002-2008	5 (22)	9 (39)			2.92	0.13
2009-2014	10 (45)	4 (18)			0.65	0.56
2015-2020	3 (11)	6 (22)			3.25	0.16
1977-1991	13 (52)		3 (12)		Ref.	
1992-2001	10 (40)		4 (16)		1.73	0.53
2002-2008	5 (22)		7 (30)		6.07	0.04
2009-2014	10 (45)		4 (18)		1.73	0.53
2015-2020	3 (11)		7 (26)		10.1	0.01
1977-1991	13 (52)			1 (4)	0.02	0.002
1992-2001	10 (40)			0 (0)	-	-
2002-2008	5 (22)			2 (9)	0.11	0.04
2009-2014	10 (45)			4 (18)	0.11	0.01
2015-2020	3 (11)			11 (41)	Ref.	
1977-1991		8 (32)	3 (12)		0.32	0.20
1992-2001		11 (44)	4 (16)		0.31	0.25
2002-2008		9 (39)	7 (30)		0.67	0.59
2009-2014		4 (18)	4 (18)		0.86	0.86
2015-2020		6 (22)	7 (26)		Ref.	
1977-1991		8 (32)		1 (4)	0.07	0.02
1992-2001		11 (44)		0 (0)	-	-
2002-2008		9 (39)		2 (9)	0.12	0.02

2009-2014		4 (18)		4 (18)	0.54	0.49
2015-2020		6 (22)		11 (41)	Ref.	
1977-1991			3 (12)	1 (4)	0.21	0.22
1992-2001			4 (16)	0 (0)	-	-
2002-2008			7 (30)	2 (9)	0.18	0.07
2009-2014			4 (18)	4 (18)	0.64	0.60
2015-2020			7 (26)	11 (41)	Ref.	
Number of years in cattle practice						$R^2 = 0.10$
<5	4 (14)	5 (18)			2.29	0.32
5-9	8 (40)	5 (25)			1.15	0.86
10-14	4 (22)	8 (44)			3.67	0.10
15-19	7 (37)	7 (37)			1.83	0.41
20-24	7 (41)	7 (41)			1.83	0.41
≥25	11 (55)	6 (30)			Ref.	
<5	4 (14)		7 (25)		9.63	0.02
5-9	8 (40)		4 (20)		2.74	0.30
10-14	4 (22)		5 (28)		6.87	0.06
15-19	7 (37)		4 (21)		3.14	0.25
20-24	7 (41)		3 (18)		2.36	0.41
≥25	11 (55)		2 (10)		Ref.	
<5	4 (14)			12 (43)	Ref.	
5-9	8 (40)			3 (15)	0.12	0.02
10-14	4 (22)			1 (6)	0.08	0.05
15-19	7 (37)			1 (5)	0.05	0.01
20-24	7 (41)			0 (0)	-	-
≥25	11 (55)			1 (5)	0.03	0.003
<5		5 (18)	7 (25)		Ref.	
5-9		5 (25)	4 (20)		0.57	0.53
10-14		8 (44)	5 (28)		0.45	0.32
15-19		7 (37)	4 (21)		0.41	0.30
20-24		7 (41)	3 (18)		0.31	0.19
≥25		6 (30)	2 (10)		0.24	0.15
<5		5 (18)		12 (43)	Ref.	
5-9		5 (25)		3 (15)	0.25	0.13
10-14		8 (44)		1 (6)	0.05	0.01
15-19		7 (37)		1 (5)	0.06	0.02
20-24		7 (41)		0 (0)	-	-
≥25		6 (30)		1 (5)	0.07	0.03
<5			7 (25)	12 (43)	Ref.	
5-9			4 (20)	3 (15)	0.44	0.36
10-14			5 (28)	1 (6)	0.12	0.07
15-19			4 (21)	1 (5)	0.15	0.11
20-24			3 (18)	0 (0)	-	-
≥25			2 (10)	1 (5)	0.29	0.35

[1] RRR = relative risk ratio; Ref. = referent; CMT = California mastitis test. [2] R^2 = coefficient of variation (the proportion of the variation in the dependent variable that is predictable from the independent variable).

How often do you recommend follow-up the DCT effect by bacteriology after calving?	Always/ almost always N (%)	Quite often N (%)	Less often N (%)	Never/ almost never N (%)	RRR[1]	P-value[3]
Year of degree[2]						$R^2 = 0.11$
1977-1991	0 (0)	5 (22)			-	-
1992-2001	4 (17)	2 (9)			Ref.	
2002-2008	0 (0)	1 (5)			-	-
2009-2014	1 (5)	1 (5)			2.00	0.68
2015-2020	0 (0)	2 (7)			-	-
1977-1991	0 (0)		13 (56)		-	-
1992-2001	4 (17)		10 (44)		Ref.	
2002-2008	0 (0)		13 (62)		-	-
2009-2014	1 (5)		7 (33)		2.80	0.40
2015-2020	0 (0)		8 (30)		-	-
1977-1991	0 (0)			5 (22)	-	-
1992-2001	4 (17)			7 (30)	Ref.	
2002-2008	0 (0)			7 (33)	-	-
2009-2014	1 (5)			12 (57)	6.86	0.11
2015-2020	0 (0)			17 (63)	-	-
1977-1991		5 (22)	13 (56)		Ref.	
1992-2001		2 (9)	10 (44)		1.92	0.49
2002-2008		1 (5)	13 (62)		5.00	0.17
2009-2014		1 (5)	7 (33)		2.69	0.41
2015-2020		2 (7)	8 (30)		1.54	0.65
1977-1991		5 (22)		5 (22)	Ref.	
1992-2001		2 (9)		7 (30)	3.50	0.22
2002-2008		1 (5)		7 (33)	7.00	0.12
2009-2014		1 (5)		12 (57)	12.0	0.04
2015-2020		2 (7)		17 (63)	8.50	0.03
1977-1991			13 (56)	5 (22)	Ref.	
1992-2001			10 (44)	7 (30)	1.82	0.41
2002-2008			13 (62)	7 (33)	1.40	0.63
2009-2014			7 (33)	12 (57)	4.46	0.04
2015-2020			8 (30)	17 (63)	5.53	0.01
Post-graduate training						$R^2 = 0.05$
Yes	4 (6)	10 (15)			Ref.	
No	1 (2)	1 (2)			0.40	0.55
Yes	4 (6)		34 (50)		Ref.	
No	1 (2)		17 (37)		2.00	0.55
Yes	4 (6)			20 (29)	Ref.	
No	1 (2)			27 (59)	5.40	0.15
Yes		10 (15)	34 (50)		Ref.	
No		1 (2)	17 (37)		5.00	0.14

Yes		10 (15)		20 (29)	Ref.	
No		1 (2)		27 (59)	13.5	0.02
Yes			34 (50)	20 (29)	Ref.	
No			17 (37)	27 (59)	2.70	0.02
Number of years in cattle practice						R^2 = 0.14
<5	0 (0)	1 (4)			-	-
5-9	1 (5)	2 (10)			Ref.	
10-14	0 (0)	0 (0)			-	-
15-19	2 (11)	2 (11)			0.50	0.66
20-24	2 (13)	1 (7)			0.25	0.42
≥25	0 (0)	5 (28)			-	-
<5	0 (0)		8 (29)		-	-
5-9	1 (5)		7 (35)		Ref.	
10-14	0 (0)		12 (75)		-	-
15-19	2 (11)		7 (39)		0.50	0.60
20-24	2 (13)		6 (40)		0.43	0.53
≥25	0 (0)		11 (61)		-	-
<5	0 (0)			19 (68)	-	-
5-9	1 (5)			10 (50)	Ref.	
10-14	0 (0)			4 (25)	-	-
15-19	2 (11)			7 (39)	0.35	0.43
20-24	2 (13)			6 (40)	0.30	0.37
≥25	0 (0)			2 (11)	-	-
<5		1 (4)	8 (29)		3.64	0.28
5-9		2 (10)	7 (35)		1.59	0.63
10-14		0 (0)	12 (75)		-	-
15-19		2 (11)	7 (39)		1.59	0.63
20-24		1 (7)	6 (40)		2.73	0.41
≥25		5 (28)	11 (61)		Ref.	
<5		1 (4)		19 (68)	47.5	0.004
5-9		2 (10)		10 (50)	12.5	0.03
10-14		0 (0)		4 (25)	-	-
15-19		2 (11)		7 (39)	8.75	0.06
20-24		1 (7)		6 (40)	15.0	0.05
≥25		5 (28)		2 (11)	Ref.	
<5			8 (29)	19 (68)	Ref.	
5-9			7 (35)	10 (50)	0.60	0.43
10-14			12 (75)	4 (25)	0.14	0.006
15-19			7 (39)	7 (39)	0.42	0.20
20-24			6 (40)	6 (40)	0.42	0.23
≥25			11 (61)	2 (11)	0.08	0.003

[1] RRR = relative risk ratio; Ref. = referent. [2] In addition, a larger proportion of veterinarian with a degree from 1992-2001 answered less often than those with a degree from 2015-2020 (RRR = 3.95, P = 0.03) who more often answered never/almost never. [3] R^2 = coefficient of variation (the proportion of the variation in the dependent variable that is predictable from the independent variable).

Supplementary Table S3. Significant associations between herd variables and responses t10.0o questions about routines for treating dairy cows with internal teat sealants (ITS) at drying-off given by farmers (n = 338) participating in a web-based questionnaire as analysed using univariable logistic or multinominal logistic regression models

Do you use ITS?	Yes N (%)	No N (%)	OR[1]	P-value[3]
Milk production, kg ECM[2]/cow/year				$R^2 = 0.05$
<9,000	2 (5)	38 (95)	0.12	0.005
9,000-11,000	28 (14)	167 (86)	0.38	0.001
>11,000	31 (31)	70 (69)	Ref.	
Milking system				$R^2 = 0.10$
AMS[1]	25 (18)	117 (82)	2.99	0.02
Tie-stall	7 (7)	98 (93)	Ref.	
Parlour	21 (27)	58 (73)	5.07	0.001
Rotary	7 (78)	2 (22)	49.0	<0.001
Combinations	1 (33)	2 (67)	7.00	0.13
Region				$R^2 = 0.06$
East Sweden	2 (4)	49 (96)	Ref.	
Norrland	5 (10)	44 (90)	2.78	0.23
Northern Middle Sweden	9 (30)	21 (70)	10.5	0.004
Småland and the islands	12 (17)	60 (83)	4.90	0.04
South Sweden	13 (30)	31 (70)	10.3	0.003
West Sweden	29 (22)	72 (78)	6.81	0.01

[1] OR = odds ratio; Ref. = referent; AMS = automatic milking system. [2] ECM = energy-corrected milk. [3] R^2 = coefficient of variation (the proportion of the variation in the dependent variable that is predictable from the independent variable).

ITS not used due to good udder health?	Yes N (%)	Not given as reason N (%)	OR[1]	P-value[2]
Bulk milk SCC[1], cells/ml				$R^2 = 0.05$
<200,000	83 (46)	99 (54)	3.61	<0.001
≥200,000	16 (19)	69 (81)	Ref.	
Milking system				$R^2 = 0.03$
AMS[1]	30 (27)	83 (73)	Ref.	
Tie-stall	41 (43)	54 (57)	2.10	0.01
Parlour	27 (47)	30 (53)	2.49	0.007
Rotary	2 (100)	0 (0)	-	-
Combinations	0 (0)	2 (100)	-	-
Number of cows/herd				$R^2 = 0.03$
<53	37 (47)	41 (53)	2.98	0.005
53-77	23 (33)	47 (67)	1.62	0.24
78-137	26 (41)	38 (59)	2.26	0.04
≥138	13 (23)	43 (77)	Ref.	

[1] OR = odds ratio; Ref. = referent; SCC = somatic cell count; AMS = automatic milking system. [2] R^2 = coefficient of variation (the proportion of the variation in the dependent variable that is predictable from the independent variable).

Supplementary Table S4. Significant associations between veterinary variables and responses to questions about advice on treating dairy cows with internal teat sealants (ITS) at drying-off given by veterinarians (n = 130) participating in a web-based questionnaire as analysed using univariable logistic or multinominal logistic regression models

How often do you prescribe ITS?	Each week N (%)	A couple of times per month N (%)	Less often N (%)	Never N (%)	RRR[1]	P-value[3]
Region[2]						$R^2 = 0.08$
East Sweden			8 (24)	20 (61)	1.60	0.54
Norrland			6 (26)	13 (56)	1.85	0.45
Northern Middle Sweden			3 (17)	12 (67)	Ref.	
Småland and the islands			6 (33)	5 (28)	4.80	0.08
South Sweden			5 (33)	3 (20)	6.67	0.05
West Sweden			8 (35)	6 (26)	5.33	0.05
East Sweden		4 (12)		20 (61)	0.09	0.005
Norrland		4 (17)		13 (56)	0.13	0.02
Northern Middle Sweden		3 (17)		12 (67)	0.11	0.02
Småland and the islands		7 (39)		5 (28)	0.60	0.57
South Sweden		7 (47)		3 (20)	Ref.	
West Sweden		7 (30)		6 (26)	0.50	0.43
East Sweden	1 (3)			20 (61)	Ref.	
Norrland	0 (0)			13 (56)	-	-
Northern Middle Sweden	0 (0)			12 (67)	-	-
Småland and the islands	0 (0)			5 (28)	-	-
South Sweden	0 (0)			3 (20)	-	-
West Sweden	3 (9)			6 (26)	6.68	0.15
East Sweden		4 (12)	8 (24)		Ref.	
Norrland		4 (17)	6 (26)		1.33	0.75
Northern Middle Sweden		3 (17)	3 (17)		2.00	0.50
Småland and the islands		7 (39)	6 (33)		2.33	0.31
South Sweden		7 (47)	5 (33)		2.80	0.22
West Sweden		7 (30)	8 (35)		1.75	0.49
East Sweden	1 (3)		8 (24)		Ref.	
Norrland	0 (0)		6 (26)		-	-
Northern Middle Sweden	0 (0)		3 (17)		-	-
Småland and the islands	0 (0)		6 (33)		-	-
South Sweden	0 (0)		5 (33)		-	-
West Sweden	3 (9)		8 (35)		2.00	0.60

East Sweden	1 (3)	4 (12)			Ref.	
Norrland	0 (0)	4 (17)			-	-
Northern Middle Sweden	0 (0)	3 (17)			-	-
Småland and the islands	0 (0)	7 (39)			-	-
South Sweden	0 (0)	7 (47)			-	-
West Sweden	3 (9)	7 (30)			1.14	0.92
Post-graduate training						$R^2 = 0.11$
Yes			21 (27)	23 (30)	Ref.	
No			15 (29)	35 (67)	0.47	0.08
Yes		30 (39)		23 (30)	Ref.	
No		2 (4)		35 (67)	0.04	<0.001
Yes	3 (4)			23 (30)	Ref.	
No	0 (0)			35 (67)	-	-
Yes		30 (39)	21 (27)		Ref.	
No		2 (4)	15 (29)		0.09	0.003
Yes	3 (4)		21 (27)		Ref.	
No	0 (0)		15 (29)		-	-
Yes	3 (4)	30 (39)			Ref.	
No	0 (0)	2 (4)			-	-
Number of mastitis cases/month						$R^2 = 0.20$
<1			2 (12)	15 (88)	0.02	0.001
1-3			7 (23)	20 (67)	0.06	0.002
4-8			13 (32)	18 (45)	0.12	0.02
9-15			11 (41)	2 (7)	Ref.	
>15			3 (19)	4 (25)	0.14	0.07
<1		0 (0)		15 (88)	-	-
1-3		3 (10)		20 (67)	0.02	<0.001
4-8		9 (23)		18 (45)	0.07	0.002
9-15		14 (52)		2 (7)	Ref.	
>15		6 (37)		4 (25)	0.21	0.12
<1	0 (0)			15 (88)	-	-
1-3	0 (0)			20 (67)	-	-
4-8	0 (0)			18 (45)	-	-
9-15	0 (0)			2 (7)	-	-
>15	3 (19)			4 (25)	Ref.	
<1		0 (0)	2 (12)		-	-
1-3		3 (10)	7 (23)		0.21	0.12
4-8		9 (23)	13 (32)		0.35	0.20
9-15		14 (52)	11 (41)		0.64	0.58
>15		6 (37)	3 (19)		Ref.	
<1	0 (0)		2 (12)		-	-
1-3	0 (0)		7 (23)		-	-

4-8	0 (0)		13 (32)		-	-
9-15	0 (0)		11 (41)		-	-
>15	3 (19)		3 (19)		Ref.	
<1	0 (0)	0 (0)			-	-
1-3	0 (0)	3 (10)			-	-
4-8	0 (0)	9 (23)			-	-
9-15	0 (0)	14 (52)			-	-
>15	3 (19)	6 (37)			Ref.	

[1] RRR = relative risk ratio; Ref. = referent. [2] In addition, a larger proportion of veterinarians in East Sweden answered never than in Småland and the islands (RRR = 7.00, P = 0.02) or West Sweden (RRR = 5.83, P = 0.02) where a larger proportion answered some time per month. [3] R^2 = coefficient of variation (the proportion of the variation in the dependent variable that is predictable from the independent variable).

Do you give advice on how to perform the treatment with ITS?	Yes N (%)	No N (%)	OR[1]	P-value[2]
Post-graduate training				$R^2 = 0.09$
Yes	49 (91)	5 (9)	Ref.	
No	11 (65)	6 (35)	5.34	0.02

[1] OR = odds ratio; Ref. = referent. [2] R^2 = coefficient of variation (the proportion of the variation in the dependent variable that is predictable from the independent variable).

How do you think ITS affects animal health and production?	Improves N (%)	No effect N (%)	Worsens N (%)	Don't know N (%)	RRR[1]	P-value[2]
Post-graduate training						$R^2 = 0.15$
Yes	49 (64)	1 (1)			Ref.	
No	18 (35)	0 (0)			-	-
Yes	49 (64)		4 (5)		Ref.	
No	18 (35)		2 (4)		1.36	0.73
Yes	49 (64)			23 (30)	Ref.	
No	18 (35)			31 (61)	3.67	0.001
Yes		1 (1)	4 (5)		Ref.	
No		0 (0)	2 (4)		-	-
Yes		1 (1)		23 (30)	Ref.	
No		0 (0)		31 (61)	-	-
Yes			4 (5)	23 (30)	Ref.	
No			2 (4)	31 (61)	2.70	0.28
Number of mastitis cases/month						$R^2 = 0.15$
<1	2 (13)	0 (0)			-	-
1-3	12 (40)	0 (0)			-	-
4-8	20 (50)	0 (0)			-	-
9-15	21 (78)	1 (4)			-	-
>15	12 (75)	0 (0)			-	-
<1	2 (13)		0 (0)		-	-
1-3	12 (40)		2 (7)		1.11	0.92
4-8	20 (50)		3 (8)		Ref.	
9-15	21 (78)		0 (0)		-	-

>15	12 (75)		1 (6)		0.55	0.63
<1	2 (13)			14 (87)	Ref.	
1-3	12 (40)			16 (53)	0.19	0.05
4-8	20 (50)			17 (42)	0.12	0.01
9-15	21 (78)			5 (19)	0.03	<0.001
>15	12 (75)			3 (19)	0.04	0.01
<1		0 (0)	0 (0)		-	-
1-3		0 (0)	2 (7)		-	-
4-8		0 (0)	3 (8)		-	-
9-15		1 (4)	0 (0)		-	-
>15		0 (0)	1 (6)		-	-
<1		0 (0)		14 (87)	-	-
1-3		0 (0)		16 (53)	-	-
4-8		0 (0)		17 (42)	-	-
9-15		1 (4)		5 (19)	-	-
>15		0 (0)		3 (19)	-	-
<1			0 (0)	14 (87)	-	-
1-3			2 (7)	16 (53)	1.41	0.72
4-8			3 (8)	17 (42)	Ref.	
9-15			0 (0)	5 (19)	-	-
>15			1 (6)	3 (19)	0.53	0.63

[1] RRR = relative risk ratio; Ref. = referent. [2] R^2 = coefficient of variation (the proportion of the variation in the dependent variable that is predictable from the independent variable).
